# Glucocorticoid therapy regulates podocyte motility by inhibition of Rac1

**DOI:** 10.1038/s41598-017-06810-y

**Published:** 2017-07-27

**Authors:** James C. McCaffrey, Nicholas J. Webb, Toryn M. Poolman, Maryline Fresquet, Cressida Moxey, Leo A. H. Zeef, Ian J. Donaldson, David W. Ray, Rachel Lennon

**Affiliations:** 1 Wellcome Trust Centre for Cell-Matrix Research, School of Biological Sciences, Faculty of Biology Medicine and Health, The University of Manchester, Manchester Academic Health Science Centre, Manchester, England; 2 Department of Paediatric Nephrology, Royal Manchester Children’s Hospital, Central Manchester University Hospitals NHS Foundation Trust, Manchester Academic Health Science Centre, Manchester, England; 3 School of Medical Sciences, Faculty of Biology Medicine and Health, The University of Manchester, Manchester Academic Health Science Centre, Manchester, England

## Abstract

Nephrotic syndrome (NS) occurs when the glomerular filtration barrier becomes excessively permeable leading to massive proteinuria. In childhood NS, immune system dysregulation has been implicated and increasing evidence points to the central role of podocytes in the pathogenesis. Children with NS are typically treated with an empiric course of glucocorticoid (Gc) therapy; a class of steroids that are activating ligands for the glucocorticoid receptor (GR) transcription factor. Although Gc-therapy has been the cornerstone of NS management for decades, the mechanism of action, and target cell, remain poorly understood. We tested the hypothesis that Gc acts directly on the podocyte to produce clinically useful effects without involvement of the immune system. In human podocytes, we demonstrated that the basic GR-signalling mechanism is intact and that Gc induced an increase in podocyte barrier function. Defining the GR-cistrome identified Gc regulation of motility genes. These findings were functionally validated with live-cell imaging. We demonstrated that treatment with Gc reduced the activity of the pro-migratory small GTPase regulator Rac1. Furthermore, Rac1 inhibition had a direct, protective effect on podocyte barrier function. Our studies reveal a new mechanism for Gc action directly on the podocyte, with translational relevance to designing new selective synthetic Gc molecules.

## Introduction

Glucocorticoid (Gc) therapy has been first-line therapy for childhood nephrotic syndrome (NS) for several decades but the mechanism of action, and target cell, remain poorly understood. NS has traditionally been viewed as a disease of immune dysfunction, and following the discovery of Gc-efficacy in the treatment of NS subtypes, strategies to identify new drug therapies have focussed on alternative immunosuppressive agents^1^. Although the majority of treatments known to be effective in NS have immunosuppressive properties, direct podocyte-specific effects have been identified as the key mechanism for some drugs including ciclosporin and the anti-CD20 antibody rituximab^2, 3^. These observations have built on extensive evidence demonstrating that the podocyte is the key target-site of injury in NS^4, 5^, and stimulated the search for podocyte-specific therapies, which may yield more efficacious drugs with an improved side-effect profile.

Understanding the effects that glucocorticoids exert on podocytes may identify key anti-proteinuric cellular mechanisms. Glucocorticoids exert cellular effects via the glucocorticoid receptor (GR). Ligand-free GR is predominantly located in the cytoplasm of cells; after binding to Gc-ligand, GR dimerizes and translocates to the nucleus to regulate transcription^6^. Phosphorylation of GR is induced by ligand-binding, and phosphorylation of serine 211 is a marker for activated GR^7^. How podocytes respond to GR activation is not understood and similarly the mechanisms underlying the onset of proteinuria are only partially understood. However, the concept of podocyte motility as a determinant of glomerular filtration barrier (GFB) function is an emerging theme in renal biology, with proteinuria representing the consequence of migratory podocytes^8, 9^. Developments in serial multiphoton imaging have allowed direct visualization of the kidney at a cellular level *in vivo* and shown that podocytes are motile along the basement membrane, and become hypermobile following renal injury^10, 11^. These observations have altered the view of the GFB from a static to a highly dynamic structure, with podocytes capable of rapidly reorganising their actin-based cytoskeleton in response to external stimuli^9, 12^.

Cell migration is a multi-step, cyclical process, usually initiated in response to extracellular cues, leading to reorganisation of the actin cytoskeleton and cell polarisation^13^. The Ras superfamily of small guanosine triphosphatases (small GTPases) are major regulators of cell migration. They comprise over 150 human members and are divided into five major branches: Ras, Rho, Rab, Ran and Arf^14^. The small GTPases Rac1 and RhoA operate antagonistically, with RhoA having a role in the initial cellular protrusion event, and Rac1 activating pathways implicated in reinforcement and stabilisation of the newly expanded protrusion^15^. Small GTPases possess intrinsic phosphatase activity and bind either guanosine triphosphate (GTP) or guanosine diphosphate (GDP). Thus, small GTPases function as molecular switches, cycling between inactive (GDP-bound) and active (GTP-bound) states. Evidence implicating Rac1 in kidney disease comes from a study examining angiotensin-II-induced podocyte injury. This study demonstrated a switch from a stationary to motile phenotype involving Rac1 in cultured mouse podocytes stably expressing the type 1 angiotensin II-receptor^16^. Babelova *et al*., examined a potential role for Rho GTPase inhibition in CKD using the 5/6 nephrectomy model of arterial hypertension and proteinuria in mice. EHT1846 (Rac1 inhibitor) markedly attenuated proteinuria as well as glomerular and tubulointerstitial damage^17^. Furthermore, mutations in a regulator of Rac1 called Rho GTPase-activating protein 24 (coded by the gene *Arhgap24*) has been associated with NS in humans^18^.

In this study, we tested the hypothesis that glucocorticoids act directly on podocytes to produce potentially clinically useful effects without involvement of the immune system. We demonstrated that Gc therapy reduces podocyte motility following exposure to proteinuria-inducing agents PAN and LPS, and this may partially explain Gc efficacy in NS. Furthermore, we provide supportive evidence that Gc reduces proteinuria-associated Rac1 overactivity, and Rac1 inhibition may have therapeutic efficacy.

## Results

### The podocyte GR-signalling pathway is functionally active *in vitro*

**Figure 1:**
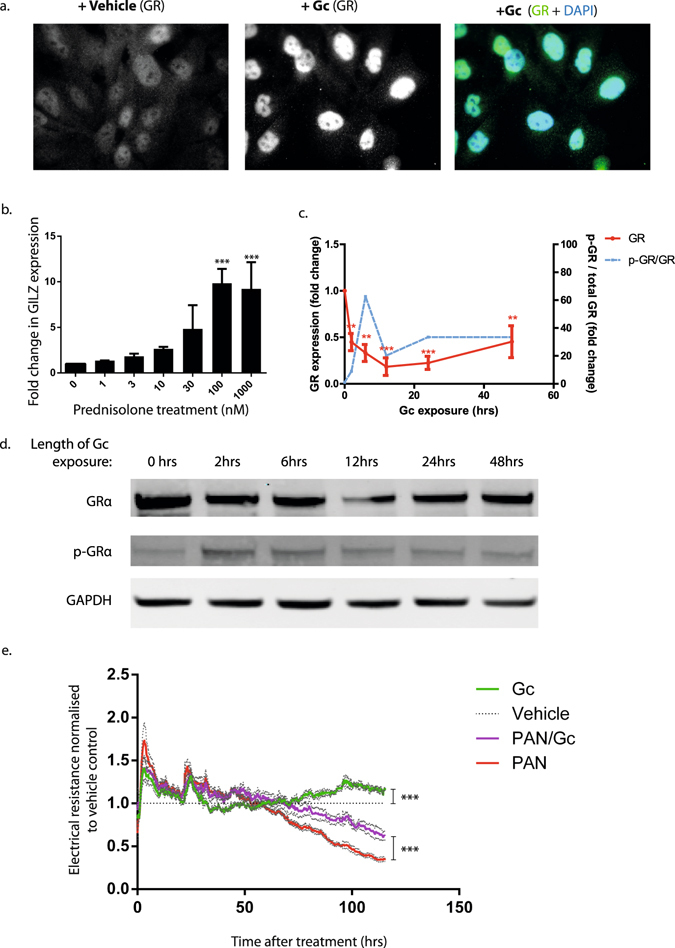
Podocyte GR expression and response to Gc exposure. (**a**) Immunofluorescence data demonstrating GR nuclear translocation following 3 hours of exposure to either vehicle or prednisolone. (**b**) Graph showing fold change in messenger RNA (mRNA) levels for a known Gc-regulated gene, glucocorticoid-induced leucine zipper (GILZ), following exposure to different concentrations of prednisolone for 6 hours. mRNA levels were quantified using real-time polymerase chain reaction (qPCR). The experiment was performed three times. Results were compared to untreated and analysed using one way ANOVA followed by Dunnet’s multiple comparisons test. *** = adjusted p value < 0.005. (**c**) Quantification of western blotting results. GR expression is reduced as early as 2 hours following prednisolone exposure. The solid red line represents the mean average GR expression and error bars represent the standard error of the mean (SEM). Results were analysed by one-way ANOVA followed by Dunnett’s multiple comparisons test. ** = adjusted p value < 0.05; *** = adjusted p value < 0.005. Blue line represents mean average of GR phosphorylated at serine residue 211 divided by total GR. No significant difference in phospho-GR levels were found. The experiment was repeated 3 times. (**d**) Podocytes express GRα as demonstrated by western blotting. Exposure to the GR-ligand prednisolone results in decreased GR expression. (**e**) Direct effects of prednisolone on a monolayer of podocytes were investigated using Electric Cell-Substrate Impedance Sensing (ECIS) which measures resistance across a monolayer of podocytes over time. Higher resistance is a surrogate marker for higher barrier integrity. Combined data from 3 separate experiments, normalised to the vehicle control is shown. Data was analysed using one way ANOVA followed by Tukey’s multiple comparison test. *** = adjusted p value < 0.0001. The grey dotted line surrounding each continuous coloured line denotes the SEM. Full-length western blots are provided in Supplementary Figure S7.

To investigate whether human immortalized wild type podocytes are directly responsive to Gc exposure, we first confirmed ligand-dependent translocation of GR into podocyte nuclei using immunofluorescence (Fig. 1a). Gc treatment also elicited a dose-dependent increase in levels of mRNA for a known Gc-regulated gene, glucocorticoid-induced leucine zipper (*GILZ*) (Fig. 1b). Podocyte exposure to the GR ligand prednisolone resulted in reduced GR expression by the 2- hour time point, a nadir of GR expression at 12 hours, followed by a gradual recovery in expression levels over the following 48 hours (Fig. 1c,d). Phosphorylation of GR at serine residue 211 peaked at the 2-hour time point following prednisolone exposure, and subsequently decreased until reaching a steady level at the 24-hour time point. These data demonstrate that the basic GR-signalling mechanism in intact in human podocytes, and these cells are capable of responding directly to Gc exposure.

### Prednisolone modulates podocyte barrier function

Having demonstrated that GR-signalling was intact in human podocytes, we investigated protective effects of prednisolone, following podocyte injury without the involvement of immune cells. We used Electric Cell-Substrate Impedance Sensing (ECIS)^19^ and the well-characterized puromycin amino nucleoside (PAN) model of podocyte injury^20, 21^. We demonstrated that well-defined actin fibres apparent in the vehicle-treated podocytes were lost in podocytes treated with 5.0 µg/mL PAN for 24 hours (Supplementary Figure S1). Furthermore, the resistance across a monolayer of podocytes treated with PAN alone markedly decreased, signifying podocyte injury (Fig. 1e). However, podocytes treated with prednisolone alone showed higher resistance than vehicle-treated cells. Interestingly, Gc exposure provided some protective effect against PAN-injury, as demonstrated by the higher resistance recorded for PAN/Gc cells compared to cells treated with PAN alone.

### Gc-regulated changes in the podocyte transcriptome

**Figure 2:**
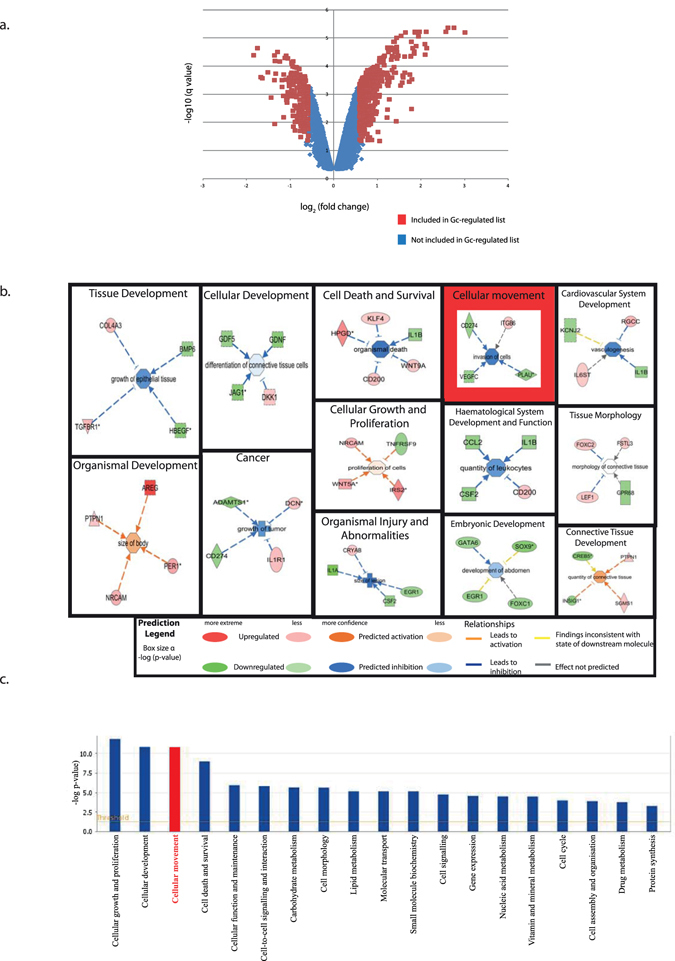
Microarray analysis of Gc-regulated genes. Microarray analysis was performed on transcripts from podocytes following 5 hour exposure to either prednisolone or vehicle. A fold-change in transcript expression of ±1.5 and a q-value of <0.05 between vehicle- and prednisolone-treated samples was considered significant, and this defined the list of Gc-regulated genes. (**a**) is a volcano plot showing the distribution of Gc-regulated transcripts. (**b**) This list of genes underwent pathway analysis using the following terms: ‘disease and disorders,’ ‘molecular and cellular functions’ and ‘physiological system development and function’ to understand the range of processes regulated by Gc. Significantly enriched terms are displayed, with the size of each box being inversely proportional to the p-value. Each displayed term has a pathway analysis example contained within the box, illustrating the relationship between transcriptional changes and effects on lower-order IPA pathway terms. (**c**) Podocyte Gc-regulated genes underwent pathway analysis using the term ‘molecular and cellular functions.’ Enriched terms are listed in order of significance. The orange line denotes a threshold level of significance corresponding to a p-value of 0.05. ‘Cellular movement’ was a term selected for further analysis and is highlighted in red.

In order to understand Gc-effects on the podocyte *in vitro*, the transcriptome of differentiated wild-type human podocytes following a 5-hour treatment with either prednisolone or vehicle was performed using whole-genome Affymetrix U133 Plus 2.0 Arrays. In order to derive a list of Gc-regulated genes, transcripts showing a fold change of >1.5 fold (either up or down) and a q-value of <0.05 were considered to be significantly altered between vehicle and Gc conditions, in line with other Gc-microarray studies^22, 23^. A volcano plot (Fig. 2a) shows the distribution of Gc-regulated transcripts in relation to the total transcript population detected. A >1.5 fold selection resulted in 606 significantly altered transcripts/probe set identifiers (corresponding to 397 unique genes: the difference is due to probe-set redundancy). 267/397 genes were upregulated, while 130/397 showed reduced expression following treatment with Gc. The list of 397 Gc-regulated genes was analysed using Ingenuity Pathway Analysis (IPA) software in order to identify key Gc regulatory pathways.

### Predicted Gc-effects on biological function

In order to understand the biological processes regulated by Gc exposure in the podocyte, the Gc-regulated list of genes derived from the microarray underwent pathway analysis using IPA. Individual genes in the Gc-regulated gene list will have one or more known biological functions identified using the IPA Knowledge Base. If enough genes with the same biological function are found in the gene list, that biological function will be considered enriched. Identifying these biological functions is key to understanding the mechanism underlying the protective effects of Gc therapy. Pathway analysis in IPA can be used to understand which diseases and general physiological functions are altered by the changes observed in the dataset. Figure 2b displays the results of pathway analysis using all terms available on IPA, viz., ‘disease and disorders,’ ‘molecular and cellular functions’ and ‘physiological system development and function’ with an example of a pathway analysis relevant to each displayed significant term. Figure 2c displays the biological functions linked to the gene expression changes ranked in order of statistical significance using the search term ‘molecular and cellular functions’. As expected, these data show that glucocorticoids regulate a wide variety of functions within the cell including growth, apoptosis and metabolism. However, unexpectedly, ‘cellular movement’ was a highly ranked term (third overall). The Gc-regulated genes involved in cell motility are listed in Supplementary Table S1. As it is already known that a hypermotile podocyte phenotype is associated with proteinuria *in vivo* ^8, 9^, this *in silico* prediction of an effect of Gc exposure on podocyte motility was intriguing, and we decided to pursue this further.

### The podocyte GR cistrome

**Figure 3:**
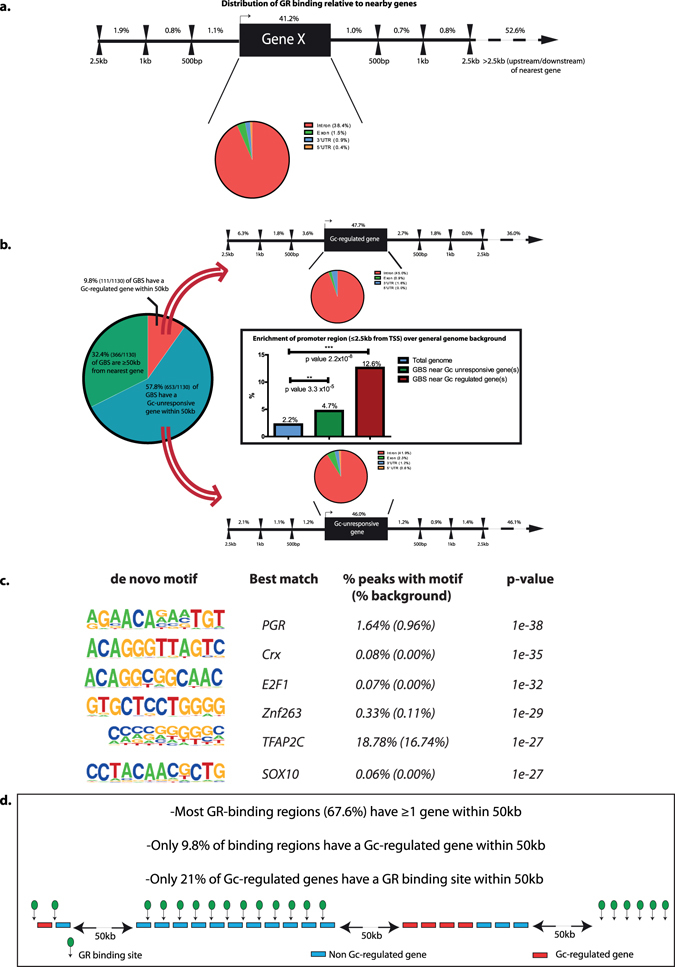
The GR cistrome. GR-binding sites (GBS) in human wild type podocytes were identified using ChIP-Seq and were analysed using the *cis*-regulatory element annotation system (CEAS). (**a**) Illustrates the percentage of GBS located in different functional genomic units. (**b**) The relation between GBS and the transcriptional output elicited by Gc-exposure identified by microarray was compared. The large pie chart shows division of GBS according to distance from Gc-regulated genes. Linked diagrams shows the percentage of GBS sites found in areas surrounding Gc –responsive and –unresponsive genes. Bar chart shows GBS enrichment in 2.5 kb promoter region over general genome background for Gc –responsive and –unresponsive genes. (**c**) Results of *de novo* motif enrichment on GR ChIP-Seq peaks using HOMER. The size of the letter for each nucleotide position relates to how dominant the base is for that position.

To understand how GR translates the Gc signal into genomic outputs, we identified GR binding sites (GBS) on a genome-wide scale in human podocytes using chromatin immunoprecipitation coupled with high throughput DNA sequencing (ChIP-Seq). In total, 1,130 GBS were identified following exposure to Gc and were distributed over important genomic features (Fig. 3a). Consistent with data from previous studies, the majority of GBS were located outside the intragenic and immediate proximal promoter region of genes^24–26^. 56.6% of the GBS observed in our dataset were located >2.5 kilobases (kb) away from the nearest transcriptional start site, with 41.2% of GBS located intragenically. We then investigated the relationship between the location of GBS and the Gc-responsive genes identified in the microarray dataset. Frequently, genes have been operationally defined as the coding sequence plus a fixed physical distance in each direction^27^. Lengths of the extensions have been from 0 to 500 kb^28, 29^, but most often of 20 kb^30–32^ or 50 kb^33–37^. For analysis, we separated GBS into 3 categories: i) GBS located >50 kb away from the coding sequence of any gene; ii) GBS located ≤50 kb away from a Gc-regulated gene (as defined by our microarray dataset); and iii) GBS located ≤50 kb away from a Gc-unresponsive gene (as defined by the microarray dataset). 32.4% of GBS were located >50 kb from the nearest gene (Fig. 3b). We then performed separate *Cis*-regulatory element annotation system (CEAS) analysis on GBS within 50 kb of a Gc-regulated gene and compared this to CEAS analysis on GBS within 50 kb of a Gc-unresponsive gene to gain insight into whether the site of GR-binding impacts gene Gc-responsiveness. This revealed that when a GBS is located within 50 kb of a Gc-responsive gene it has a higher likelihood of binding in the immediate 2.5 kb upstream promoter region. There was a greater enrichment of GBS in the 2.5 kb upstream promoter region for Gc-responsive genes compared to Gc-unresponsive genes (Fig. 3b). Although when GBS do occur within 50 kb of a Gc-responsive gene, binding within the 2.5 kb upstream region is enhanced, GBS are found within 50 kb of a Gc-responsive gene only in a minority of cases. Only 83/297 (20.9%) of Gc-regulated genes are located within 50 kb of a GBS. Motif analysis provided insight into the nucleotide sequences where GR preferentially bound. *De novo* motif enrichment showed that GR bound to transcriptional activator motifs (Fig. 3c). The most highly-significant *de novo* binding motif identified was very similar to a known progesterone receptor (PR) binding motif. This is unsurprising as PR and GR have highly homologous DNA-binding domains. A summary overview of the ChIP-Seq data is provided in Fig. 3d.

### Gc effects on podocyte motility

**Figure 4:**
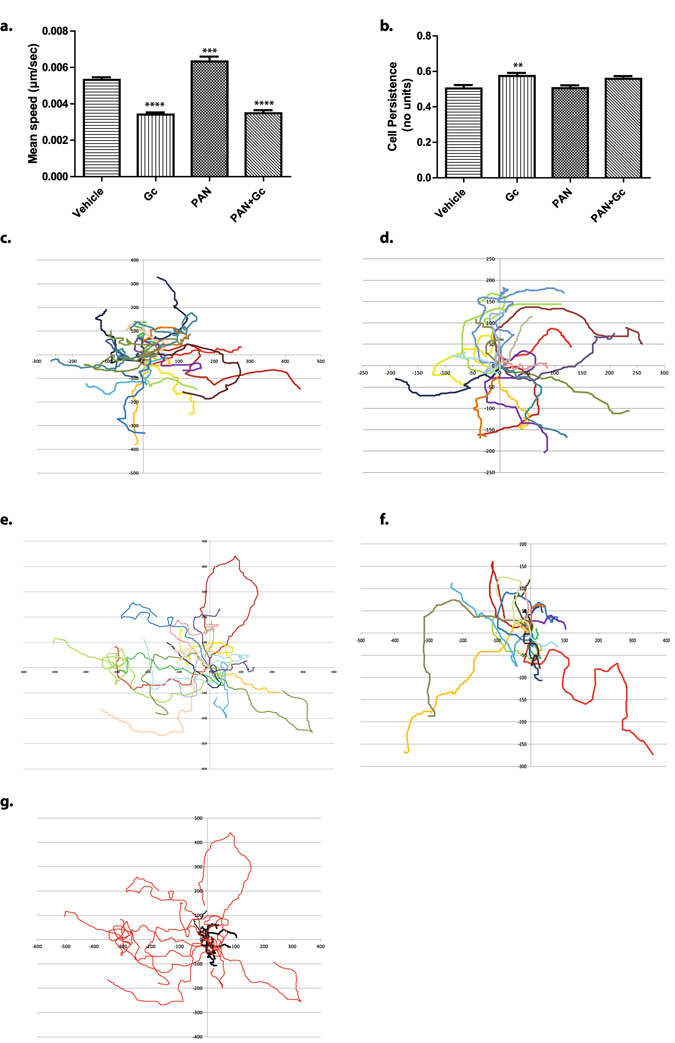
Effects of Gc and puromycin aminonucleoside (PAN) exposure on podocyte motility. Microarray analysis of Gc-regulated genes from wild-type podocytes suggested Gc exposure affected podocyte motility. In order to test this, live cell imaging of wild type podocytes was performed for a 24 hour period, beginning 24 hours following treatment. Subsequently, manual cell tracking was performed using the MTrackJ plugin on ImageJ software to allow analysis of podocyte speed and cell directional persistence. Each experiment consisted of tracking 120 cells per condition. The experiment was performed 3 times. (**a**) Quantifies mean podocyte speed over the 24 hour period. (**b**) Quantifies podocyte directional persistence. Rose plots were created for each condition to visualize the path travelled for 20 cells (the path of each cell is marked with a different colour). The x- and y-axis refer to x,y coordinates of each cell over time generated by the cell tracking software: (**c**) vehicle-treated; (**d**) Gc-treated; (**e**) PAN-treated; (**f**) PAN + Gc-treated. (**g**) is a rose plot comparing the movement of 10 PAN-treated podocytes (red) compared with 10 podocytes treated with PAN + Gc (black) over the 24 hour period. Results were compared to vehicle-treated and analysed by one-way ANOVA followed by Dunnett’s multiple comparisons test. ** = adjusted p value 0.091; *** = adjusted p value 0.0003; **** = adjusted p value < 0.0001. N.S. = not significant. Error bars represent standard error of the mean.

Gc-exposure reduced the mean speed of podocytes over the 24 hour period by approximately 36%, from 0.0053 µm/sec (vehicle-treated) to 0.0034 µm/sec (Gc-treated) (Fig. 4a). The hypermobile PAN-treated podocytes also responded to Gc-treatment by reducing speed from 0.0063 m/sec (PAN-treated) to 0.0035 µm/sec (PAN + Gc-treated). To determine if Gc-treatment also affected the directional persistence of podocyte movement (the ratio of the distance between the starting and final point of each track travelled by each podocyte, ie, Euclidean distance, compared to the total distance travelled) was calculated. A cell persistence ratio of 1 corresponds to a cell that travels in a perfectly straight line during the whole period of imaging, while a ratio tending towards 0 would imply a cell constantly changing direction. A very modest, but statistically significant increase in podocyte persistent movement from 0.50 (vehicle-treated) to 0.57 (Gc-treated).was observed (Fig. 4b) In order to visualise movement patterns of podocytes in the different treatment conditions, rose plots were constructed, which displayed movement of individual cells over the imaging period using the x,y coordinates generated by the tracking software (Fig. 4c–f). To ascertain Gc-effects on cell motility in the context of another podocyte-damaging agent, the live cell imaging was repeated using lipopolysaccharide (LPS) instead of PAN (Supplemental Figure S2). We found a reduction in podocyte motility following Gc-treatment (Supplementary Figure 2A). Here, a 51% decrease in podocyte speed was observed: from 0.021 µm/sec (vehicle-treated) to 0.010 µm/sec (Gc-treated). The hypermobile LPS-treated podocytes displayed reduced motility when LPS was co-administered with Gc. Also, Gc-treated podocytes (cell persistence ratio 0.58) showed a small increase in persistence compared to vehicle-treated podocytes (0.51), and LPS-treated podocytes displayed reduced directional movement (0.43).

To validate the effect that Gc exerts on podocyte motility, we performed a scratch-wound assay. The relative change in scratch wound area was quantified at 2 hours post-wound and 4 hours post-wound for vehicle- and Gc-treated podocytes. Supplementary Figure S3 shows that at both time points, cells treated with vehicle showed a greater efficiency (0.34 for vehicle vs 0.16 for Gc-treated at T = 2 hrs; 0.71 for vehicle vs 0.44 for Gc-treated at T = 4 hrs, p value =< 0.0001). This confirms the prominent hypomobile-inducing effect of Gc on podocytes.

**Figure 5:**
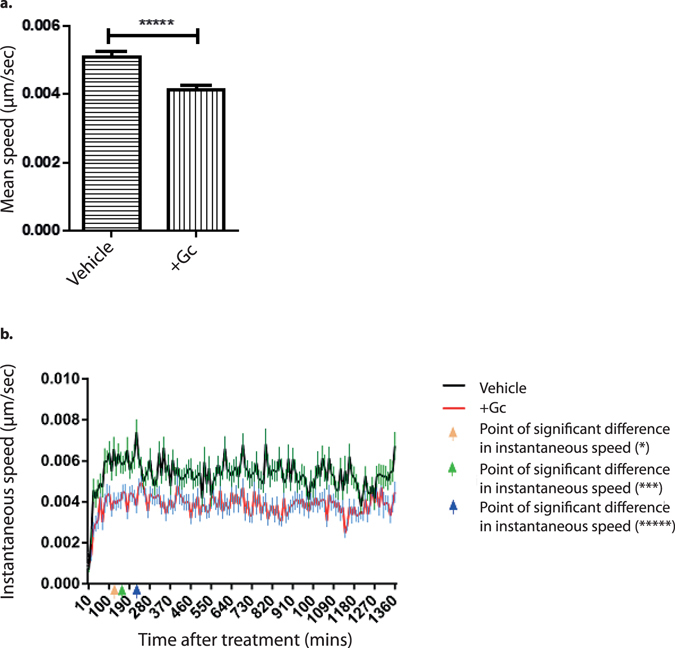
Early effects of Gc on podocyte motility. To analyse the length of time taken for Gc to affect podocyte speed, live cell imaging of wild type podocytes was performed in the 24 hour period following treatment. Each experiment consisted of manually tracking 120 cells per condition using ImageJ software. The experiment was performed twice. (**a**) Shows the mean cell speed during the whole period of imaging. (**b**) Shows the instantaneous speed for the podocytes during the 24 hour period. Coloured arrows on the X-axis refer to the level of significance of the difference between the means of cell speed between vehicle- and Gc-treated cells. The first time of significant difference is shown by the arrow at 120 minutes. Results were analysed by two-way repeated measures ANOVA followed by Šídák’s multiple comparisons test. * = adjusted p value 0.0134; *** = adjusted p value 0.0004; **** = adjusted p value < 0.0001. Error bars represent standard error of the mean.

To determine the time taken for Gc to have an effect on podocyte motility, the speed of vehicle- and Gc-treated podocytes were compared during the first 24 hours following treatment. The instantaneous speed of podocytes at each time point was measured (Fig. 5). The first statistically-significant difference in the mean speed of the vehicle- and Gc-treated podocyte populations occurred 120 minutes following treatment (p-value 0.013). The statistical significance of this difference had increased at the 180-minute time-point (p value 0.0004) and again at the 220 minute time-point (p value < 0.0001).

### Gc effects on Rac1 and RhoA

**Figure 6:**
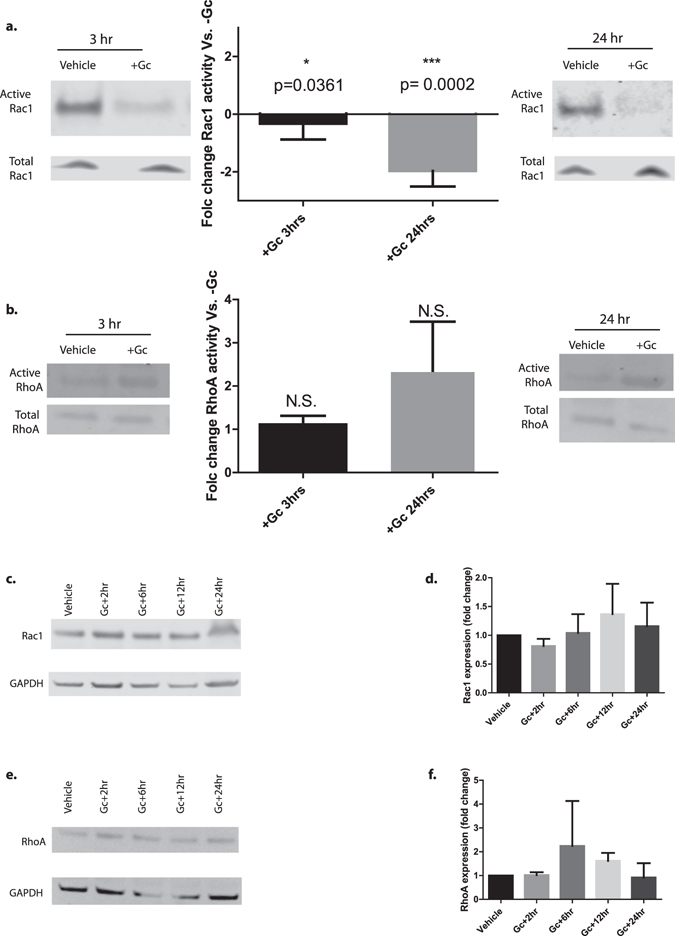
Gc effects on Rac1 and RhoA activity and expression. To understand effects of Gc exposure on the activity of two key regulators of cell motility, Rac1 and RhoA, pull-down assays using beads specific for the active form of these proteins was performed. This allowed quantification of the active protein: total protein. (**a**) Demonstrates Rac1 activity at 3 hours and 24 hours following Gc exposure. (**b**) Demonstrates RhoA activity at 3 hours and 24 hours following Gc exposure. Results were analysed using the nonparametric Mann-Whitney U test: vehicle-control treated cells were compared with Gc-treated cells at the 3 hour time point, and separate vehicle-control treated cells were compared with Gc-treated cells at the 24 hour time point for each experiment. The experiment was performed at least six times. N.S. = not significant. Western blot (**c**) and quantification (**d**) of total Rac1 expression following Gc exposure (n = 3), and western blot (**e**) and quantification (**f**) of RhoA expression (n = 2), showed no significant change in expression following analysis using one way ANOVA and Dunnett’s multiple comparisons test for any time point measured. Error bars represent standard error of the mean. Full length western blots are provided in Supplementary Figure S8.

To understand if Gc action affected the activity of either the GTPases Rac1 or RhoA, pull-down assays using beads specific for the active, GTP-bound protein were performed, and the active protein component was normalized to the total (inactive plus active) protein content. Data were obtained at the 3-hour time point as this corresponded to the approximate time at which differences in cellular motility following Gc-exposure were first observed in the live-cell imaging experiment, and also at the 24 hour time point. Gc reduced Rac1 activity at both the 3 hour time point and 24 hour time point compared to vehicle-treated cells harvested at the same time point (Fig. 6a). Although RhoA showed a trend towards increased activity following Gc exposure, this was not statistically significant (p value 0.9 at both 3 hour time point and 24 hour time point). Gc exposure did not significantly affect total Rac1 or RhoA protein expression (Fig. 6c–f). As Gc-exposure reduced Rac1 activity, we investigated whether the converse was true by performing pull-down assays for active Rac1 following 24 hours of PAN-exposure. Indeed, PAN-damaged podocytes did show an increase in Rac1 activity following treatment (Supplemental Figure S4).

### Protective effects of Rac1 inhibition

**Figure 7:**
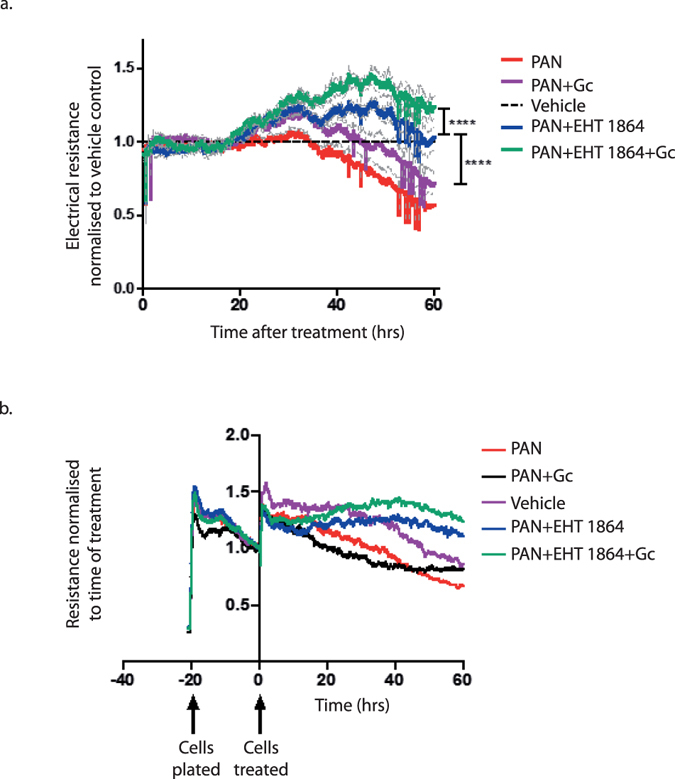
Effect of Rac1 inhibition on podocyte barrier function. The effects of Rac1 inhibition with the small molecule EHT 1864 on electrical resistance across a monolayer of podocytes was examined using electrical cell-substrate impedance sensing (ECIS). Here, electrical resistance is used as a surrogate marker for cell barrier integrity, with higher electrical resistance implying higher barrier integrity. (**a**) Shows the combined results of three experiments normalised to the vehicle control. Each experiment consisted of 3 plated wells of cells per condition, with each experiments lasting for 60 hours following treatment. The experiment was performed three times. Measured electrical resistances during the final 30 minutes of the experiment for each condition were used for final statistical analysis. Statistical analysis was performed using one-way ANOVA followed by Tukey’s multiple comparisons test. **** = p value < 0.0001. The thin grey lines represent standard error of the mean. (**b**) shows an example of a single experiment, with the resistance on the y-axis normalised to measured resistance at the time of treatment for each condition.

Since we found Gc exposure resulted in reduced podocyte speed and reduced activity of the pro-migratory regulator Rac1, we investigated whether Rac1 inhibition had an effect on podocyte motility (Supplementary Figure S5). Live cell imaging followed by cell tracking was performed and this demonstrated that Rac1 inhibition resulted in a marked 93% reduction in podocyte motility (mean speed of vehicle-treated podocytes = 0.007 µm/sec compared to 4.7 × 10^−4^ µm/sec for cells treated with the specific Rac1 inhibitor EHT 1864, p =< 0.0001). As there is an established association between a hypermobile podocyte and proteinuria^11^, we investigated whether inhibiting Rac1 with the small molecule EHT 1864 would have protective effects on podocytes against PAN-induced injury. Podocytes simultaneously treated with PAN and EHT 1864 had higher electrical resistance compared to podocytes treated with PAN alone (p value < 0.0001) (Fig. 7). Also, the electrical resistance across a podocyte monolayer exposed to PAN was higher when EHT 1864 and Gc were co-administered compared to when either Gc or EHT 1864 were used as single treatments (p value < 0.0001). These data demonstrate that Rac1 inhibition reduces PAN-induced barrier dysfunction, and augments the protective effect of Gc exposure.

## Discussion

The mechanisms underlying the ability of glucocorticoids to protect podocytes from proteinuric stimuli are poorly understood. We first demonstrated that the basic GR-signalling pathway is active in podocytes, and thus these cells can respond directly to Gc-exposure, without immune system intermediaries. To provide insight into mechanisms underlying these direct Gc-effects, we identified Gc-regulated genes using microarray analysis. Analysis of the Gc-regulated transcriptome identified podocyte motility as an enriched IPA pathway term. The Gc-induced reduced podocyte motility predicted *in silico* was confirmed *in vitro* by live-cell imaging. We demonstrated that Gc-exposure also reduced activity of the pro-migratory small GTPase Rac1, and that Rac1 inhibition reduced barrier dysfunction. For hypothesis-generation, we identified direct Gc-gene targets using a short exposure (5 hours) of the GR-agonist prednisolone to differentiated (14 day) human podocytes. Cheng *et al*., previously identified Gc-targets using a prolonged 3 day exposure of the GR-agonist dexamethasone to minimally differentiated (2 day) human podocytes, and similarly identified cellular motility as an enriched gene ontology term^22^.

Analysis of the GR-binding pattern on a genome-wide scale further re-enforced the ability of podocytes to respond directly to Gc-exposure and provided insight into how GR signalling elicits transcriptional output. Identifying GBS using ChIP-Seq revealed that a large proportion of GR binding events occurred significant distances away from the gene coding region. We created a summary of locations of GBS identified in our data compared with other studies (Supplemental Figure S6). These data raise the possibility that GR acts through long-range mechanisms, or that many binding events are opportunistic and do not necessarily affect transcriptional output. Indeed, long-range looping interactions between regulatory sequences and their target genes have been analysed using chromosome conformation-capture (3 C)-based techniques, and identified chromatin interactions that span a linear genomic distance from several hundred base pairs to over 1 million base pairs^38^. In a similar manner, Wilms’ tumour 1 (WT1) ChIP-Seq experiments have shown that around 45% of WT1 genomic binding sites are located more than 50 kb away from transcription start sites^39–41^. This suggests WT1 regulates gene expression via both proximal and distal regulatory elements in an analogous manner to GR.

We subsequently demonstrated using *in vitro* live-cell imaging that podocytes had reduced motility following exposure to Gc, and Gc reduced the motility of podocytes exposed to two proteinuria-inducing agents: PAN and LPS. These observations raise the possibility that some of the beneficial effects of Gc during periods of proteinuria may be through directly preventing the increased podocyte motility associated with renal disease. The small GTPase Rac1 has already been identified as a major regulator of cellular motility and has been implicated in the development of proteinuria^42^. Active Rac1 promotes the formation of lamellipodia that drive the motility of diverse cell types^43^, and Rac1 and RhoA are mutually inhibitory^44^. We therefore proceeded to investigate Gc effects on the activity of Rac1 and RhoA to gain mechanistic insight.

Small GTPase activity assays revealed that Gc exposure reduced (pro-migratory) Rac1 activity, and showed a trend to increase the activity of RhoA. Previously, small GTPase activity in murine podocytes was investigated following exposure to dexamethasone for 30 minutes, cultured in fresh medium for a further 3 days, before cells were harvested for analysis^21^. The group found a statistically significant increase in RhoA activity in Gc-treated cells, but no difference in Rac1 activity. Discrepancies between these data and ours may be due to the GR ligand investigated (dexamethasone compared to prednisolone), the podocyte cell line used (murine compared to human) or length of time of Gc exposure. As our data suggested a prominent Gc-effect on reducing Rac1 activity consistent with the anti-migratory effect of Gc we had observed in the live-cell imaging experiments, we confirmed that damaging podocytes with PAN-exposure increased Rac1 activity.

We also investigated Gc effects on the pattern of podocyte migration. We found a small increase in directionally persistent cell movement in podocytes treated with Gc (ie, podocytes exposed to Gc displayed less frequent changes in direction of movement). Weiger *et al*., demonstrated that the directionality of 2 dimensional cell motion clearly distinguishes benign and tumorigenic cell lines, with tumorigenic cell lines harbouring less directed, more random motion^45^. The data of LPS treated podocytes displaying increased motility and lower directional persistence, which was reversed following Gc exposure to a more directionally persistent, lower-speed phenotype gave us confidence that Gc was reducing Rac1 activity. Rac1 inhibition has previously been shown to reduce proteinuria^17, 46^. We therefore returned to ECIS to investigate whether Rac1 inhibition with EHT 1864 had any direct protective effect on podocytes undergoing acute damage with PAN *in vitro*. ECIS experiments demonstrated that EHT 1864 did indeed increase the electrical resistance across the PAN-treated podocyte cell layer compared to podocytes treated with PAN alone.

Direct, podocyte-specific, effects of medication frequently used in current clinical practice for the treatment of NS have already been established^2^. In a similar manner, it is possible that Gc efficacy in NS results from direct effects on the podocyte, and does not rely on the potent immunosuppressive action of Gc. Specifically, promotion of a hypomobile podocyte phenotype and effects on the Rac1 pathway may be important. As physiological podocyte motility may have a role in the normal functioning of the GFB, and Rac1 has roles in a range of biological processes, drugs affecting cell motility must be subtly targeted. Any future *in vitro* or animal studies examining the potential therapeutic benefits of Rac1 inhibition in reducing the acute-onset proteinuria observed in NS must be careful to ensure robust toxicology screening is performed, although the possibility of anti-proteinuria medication targeted at reducing disease-associated podocyte hypermotility is an intriguing possibility.

## Concise Methods

### Electric Cell Substrate Impedance Sensing (ECIS)

Time course analysis and resistance measurement was performed using an automated cell monitoring system, Electrical Cell-Substrate Impedance Sensing (ECIS 1600R, Applied Biophysics), as used in previous studies of podocyte function. Differentiated podocytes were seeded at a density 25,000 cells per well onto 8W10E + arrays (Applied Biophysics) coated with 10 mM cysteine and 10 µg/mL fibronectin. Each experiment measured the resistance in two 8W10E + arrays, each 8W10E + array contained five wells. The electrical resistance in each well was measured using ten electrodes. The resistance was measured at regular time intervals of 30 seconds.

### Microarray analysis

Fully differentiated human wild type podocytes cultured in RPMI 1640 medium were treated with 1 µM prednisolone or an equal volume (0.001%,v/v) of methanol as a vehicle control for 5 hours at 37 °C. Total cellular RNA was isolated using the RNeasy mini kit with on-column DNase I digestion (Qiagen). RNA integrity was assessed by a Nanodrop ND100 ultralow volume-spectrophotometer (Lab Tech) followed by a 2100 Bioanalyser (Agilent). Only samples with an absorbance 260 nm/280 nm ratio 1.9–2.1 were processed further. Total RNA from each sample was used to prepare biotinylated fragmented complementary RNA according to the GeneChip^®^ Expression Analysis Protocol (Affymetrix). GeneChip^®^ Human Genome U133A Plus 2.0 Array were hybridised and scanned using the GeneArray^®^ 2500, according to the GeneChip^®^ Expression Analysis Protocol (Affymetrix). Treatments were done in triplicates and the same batch of microarrays was used for all treatments. Technical quality control and outlier analysis was performed with dChip (V2005) (www.dchip.org)^47^, using the default settings. Background correction, quantile normalization, and gene expression analysis were performed using RMA in Bioconductor^48^. To establish relationships and compare variability between patients, principal components analysis (PCA) was used since this method is able to reduce the effective dimensionality of complex gene-expression space without significant loss of information^49^. PCA was performed with Partek Genomics Solution (version 6.5, Copyright 2010, Partek Inc.) Differential expression analysis was performed using Limma using the functions lmFit and eBayes^50^. Gene lists of differentially expressed genes were controlled for false discovery rate (fdr) errors using the method of q value^51^. Probsets were selected for further analysis if the fold-change was >1.5 and q value < 0.05. Pathway analysis was performed using Ingenuity^®^ Pathway Analysis software.

### Live cell imaging

Differentiated wild type podocytes were seeded at a density of 5,000 cell/mL per well in a 24 well cell culture cluster plate (Costar). Two wells were used for each treatment condition. Cells were then filmed using an AS MDW live cell imaging system (Leica) with a 5×/NA 0.15 HC Plan Fluotar air objective (magnification 1.5×). Point visiting was used to allow multiple positions to be imaged within the same time course, and cells were maintained at 37 °C and 5% (vol/vol) CO_2_. Images were collected using a Coolsnap HQ camera, and six movies (3 movies per well) were generated for each condition. To assess cell migration, the speed and directionality of 120 cells per condition (20 cells per movie) was measured using the MTrackJ plug-in of ImageJ. Cell tracking was performed over a 24-hour period. The manual cell tracking of the LPS and PAN experiments were performed by different individuals to reduce the risk of observer-bias.

### Rac1 activity assay

Active Rac1 was affinity purified from lysates using an effector pull-down approach with GST-PAK beads. Cells were serum starved for 24 hours before treatment. At the relevant time, cells were lysed in ice-cold lysis buffer [20 mM Hepes pH 7.5, 140 mM NaCl, 1% (v/v) Igepal, 4 mM EDTA, 4 mM EGTA, 0.5% (wt/vol) sodium deoxycholate, 10% (vol/vol) glycerol] supplemented with EDTA-free protease inhibitor tablets (Roche). Lysates were clarified by centrifugation at 12,000 g, 4 °C for 5 minutes prior to snap-freezing in liquid nitrogen to preserve GTPase-activity while other batched samples were processed. Thawed lysates were then incubated with 20 µg GST-PAK beads (Cytoskeleton) for 1 hour at 4 °C. Beads were washed three times with ice-cold lysis buffer, and active Rac1 was eluted off beads by addition of Laemmli reducing sample buffer. For each condition, equal volumes of GTP-Rac1 eluted from the GST-PAK beads, and equal volume of ‘total’ extract obtained prior to snap-freezing were resolved by SDS-PAGE and analysed by Western blotting. The ratio between GTP-Rac1 and total Rac1 was quantified to determine the Rac1 activation state.

### RhoA activity assay

Active RhoA was affinity purified from lysates using an effector pull-down approach with Rhotekin RBD beads. Cells were serum starved for 24 hours before treatment. At the relevant time, cells were lysed in ice-cold lysis buffer[50 mM Tris pH 7.5, 10 mM MgCl_2_, 0.5 M NaCl, and 2% (vol/vol) Igepal] supplemented with protease inhibitor cocktail (Cytoskeleton). Lysates were clarified by centrifugation at 10,000 g, 4 °C for 1 minute prior to snap-freezing in liquid nitrogen to preserve GTPase-activity while other batched samples were processed. Thawed lysates were then incubated with 50 µg Rhotekin RBD beads (Cytoskeleton) for 1 hour at 4 °C. Beads were washed once with ice-cold wash buffer(25 mM Tris pH 7.5, 30 mM MgCl_2_, 40 mM NaCl), and active RhoA was eluted off beads by addition of Laemmli reducing sample buffer. For each condition, equal volumes of GTP-RhoA eluted from the Rhotekin-RBD beads, and equal volume of ‘total’ extract obtained prior to snap-freezing were resolved by SDS-PAGE and analysed by Western blotting. The ratio between GTP-RhoA and total RhoA was quantified to determine the RhoA activation state.

### Protein and RNA analysis

See Supplemental Methods for protocols on Western blotting, immunofluorescence and real-time PCR.

### Antibodies for immunofluorescent and western blotting studies

Antibodies were used against GR (24050-1-AP, Proteintech), phosphorylated GR (serine 211) (4161, Cell Signaling), Rac1 (610651, BD Biosciences), RhoA (ARH03, Cytoskeleton), GAPDH (G8795, Sigma-Aldrich). Secondary antibodies conjugated to Alexa Fluor 680 (A21109, Invitrogen) and Alexa Fluor 800 (A11001, Invitrogen) were used for western blotting. A secondary antibody conjugated to Cy2 (711225152, Jackson immunoresearch laboratories) was used for immunofluorescence.

### Cell culture and GR-agonist

#### Cell culture

Human wild type podocytes: a conditionally immortalized human podocyte cell line developed by transfection with the temperature-sensitive SV40-T gene, which proliferate at 33 °C, but at 37 °C enter growth arrest and express markers of differentiated *in vivo* podocytes.1 Obtained from M. Saleem, University of Bristol, UK. This cell line was derived from a nephrectomy specimen from a 3-yr-old child with glomerulosclerosis, but no primary glomerular disease^52^. This is established as the most widely-used human podocyte cell line^53, 54^. Conditionally immortalized podocytes were grown in RPMI-1640 medium with L-glutamine and NaHCO3 (R8758) supplemented with 10% (v/v) foetal calf serum (Life Technologies), 1% (v/v) insulin-transferrin-selenium (Life Technologies, 41400045), and 1% penicillin-streptomycin (P0781-stock 10,000 units penicillin and 10 mg streptomycin per mL). Proliferating podocytes were cultured at 33 °C in a 5% (v/v) CO2 humidified incubator, and passaged when confluent, by detachment with Trypsin-EDTA solution (0.5 g porcine trypsin and 0.2 g EDTA per litre of Hanks’s balanced salt solution with phenol red), and reseeded in fresh tissue culture flasks. Podocytes underwent 10-14 days differentiation at 37 °C before use. Unless otherwise stated, glucocorticoid (Gc)-treatment refers to 1 µM prednisolone (Sigma-Aldrich) dissolved in methanol.; vehicle-treatment refers to administration of an equal, 0.001% (v/v) amount of methanol alone. The Ras-related C3 botulinum toxin substrate 1 (Rac1) inhibitor EHT 1864 was used at a dose of 30 µM.

#### GR ligand

We were unable to identify any *in vitro* studies involving podocytes where prednisolone (the GR-ligand used most frequently clinically in the UK for NS) was used. Key studies investigating the effects of dexamethasone on cells *in vitro* have used a wide range of concentrations from 0.01 µM^21^ to 100 µM^55^. However, the most commonly used concentration is 100 nM (0.1 µM)^25, 56–58^. Pharmacokinetic studies have identified the mean peak plasma concentration of prednisolone in children with NS to be between 0.1 µM and 3 µM^59, 60^. *In vivo*, it is unclear how the plasma concentration of prednisolone relates to the Gc concentration of the ultrafiltrate surrounding the podocyte, and how the 11 beta-hydroxysteroid dehydrogenase system affects this. As the most common *in vitro* dose of dexamethasone used in the literature is 0.1 µM, and dexamethasone has been shown to be a ten-fold more potent GR-agonist than prednisolone in luciferase transactivation assays^61^, a dose of 1 µM prednisolone was used in the current study, unless otherwise stated.

#### Rac1 inhibition

EHT 1864 maintains Rac1 in an inactive state and a dose of 30 µM was used, consistent with other studies^62^.

#### ChIP-Seq

See Supplemental methods.

### Data Availability

The microarray and ChIP-Seq data have been submitted to ArrayExpress (microarray: E-MTAB-5235, ChIP-Seq: E-MTAB-5238).

## Electronic supplementary material

Supplementary Information

## References

[1] Greenbaum LA, Benndorf R, Smoyer WE (2012). Childhood nephrotic syndrome–current and future therapies. Nat Rev Nephrol.

[2] Faul C (2008). The actin cytoskeleton of kidney podocytes is a direct target of the antiproteinuric effect of cyclosporine A. Nat. Med..

[3] Fornoni A (2011). Rituximab targets podocytes in recurrent focal segmental glomerulosclerosis. Sci. Transl. Med..

[4] Patrakka J, Tryggvason K (2009). New insights into the role of podocytes in proteinuria. Nat Rev Nephrol.

[5] Hinkes BG (2007). Nephrotic syndrome in the first year of life: two thirds of cases are caused by mutations in 4 genes (NPHS1, NPHS2, WT1, and LAMB2). Pediatrics.

[6] Oakley RH, Cidlowski JA (2013). The biology of the glucocorticoid receptor: new signaling mechanisms in health and disease. J. Allergy Clin. Immunol..

[7] Wang Z, Frederick J, Garabedian MJ (2002). Deciphering the phosphorylation “code” of the glucocorticoid receptor *in vivo*. J. Biol. Chem..

[8] Mundel P, Reiser J (2010). Proteinuria: an enzymatic disease of the podocyte?. Kidney Int..

[9] Kistler AD, Altintas MM, Reiser J (2012). Podocyte GTPases regulate kidney filter dynamics. Kidney Int..

[10] Peti-Peterdi J, Sipos A (2010). A high-powered view of the filtration barrier. Journal of the American Society of Nephrology: JASN.

[11] Hackl MJ (2013). Tracking the fate of glomerular epithelial cells *in vivo* using serial multiphoton imaging in new mouse models with fluorescent lineage tags. Nat. Med..

[12] Chen S (2010). Podocytes require the engagement of cell surface heparan sulfate proteoglycans for adhesion to extracellular matrices. Kidney Int..

[13] Ridley AJ (2001). Rho GTPases and cell migration. J Cell Sci.

[14] Wennerberg K, Rossman KL, Der CJ (2005). The Ras superfamily at a glance. J Cell Sci.

[15] Machacek M (2009). Coordination of Rho GTPase activities during cell protrusion. Nature.

[16] Hsu HH (2008). Mechanisms of angiotensin II signaling on cytoskeleton of podocytes. J. Mol. Med. (Berl.).

[17] Babelova A (2013). Activation of Rac-1 and RhoA contributes to podocyte injury in chronic kidney disease. PLoS One.

[18] Akilesh S (2011). Arhgap24 inactivates Rac1 in mouse podocytes, and a mutant form is associated with familial focal segmental glomerulosclerosis. The Journal of clinical investigation.

[19] Hausmann R (2010). Electrical forces determine glomerular permeability. Journal of the American Society of Nephrology: JASN.

[20] Kim YH (2001). Podocyte depletion and glomerulosclerosis have a direct relationship in the PAN-treated rat. Kidney Int..

[21] Ransom RF, Lam NG, Hallett MA, Atkinson SJ, Smoyer WE (2005). Glucocorticoids protect and enhance recovery of cultured murine podocytes via actin filament stabilization. Kidney Int..

[22] Cheng X, Zhao X, Khurana S, Bruggeman LA, Kao HY (2013). Microarray analyses of glucocorticoid and vitamin D3 target genes in differentiating cultured human podocytes. PLoS One.

[23] Wu DY, Ou CY, Chodankar R, Siegmund KD, Stallcup MR (2014). Distinct, genome-wide, gene-specific selectivity patterns of four glucocorticoid receptor coregulators. Nuclear receptor signaling.

[24] Yu CY (2010). Genome-wide analysis of glucocorticoid receptor binding regions in adipocytes reveal gene network involved in triglyceride homeostasis. PLoS One.

[25] Polman JA (2012). A genome-wide signature of glucocorticoid receptor binding in neuronal PC12 cells. BMC Neurosci..

[26] John S (2011). Chromatin accessibility pre-determines glucocorticoid receptor binding patterns. Nat. Genet..

[27] Rodriguez-Fontenla C, Calaza M, Gonzalez A (2014). Genetic distance as an alternative to physical distance for definition of gene units in association studies. BMC Genomics.

[28] Zhang K, Cui S, Chang S, Zhang L, Wang J (2010). i-GSEA4GWAS: a web server for identification of pathways/gene sets associated with traits by applying an improved gene set enrichment analysis to genome-wide association study. Nucleic Acids Res..

[29] Wang K, Li M, Bucan M (2007). Pathway-based approaches for analysis of genomewide association studies. Am. J. Hum. Genet..

[30] Chen LS (2010). Insights into colon cancer etiology via a regularized approach to gene set analysis of GWAS data. Am. J. Hum. Genet..

[31] Holmans P (2009). Gene ontology analysis of GWA study data sets provides insights into the biology of bipolar disorder. Am. J. Hum. Genet..

[32] Eleftherohorinou H, Hoggart CJ, Wright VJ, Levin M, Coin LJ (2011). Pathway-driven gene stability selection of two rheumatoid arthritis GWAS identifies and validates new susceptibility genes in receptor mediated signalling pathways. Hum. Mol. Genet..

[33] Liu JZ (2010). A versatile gene-based test for genome-wide association studies. Am. J. Hum. Genet..

[34] Nyholt DR (2012). Genome-wide association meta-analysis identifies new endometriosis risk loci. Nat. Genet..

[35] Tang W (2012). Genetic associations for activated partial thromboplastin time and prothrombin time, their gene expression profiles, and risk of coronary artery disease. Am. J. Hum. Genet..

[36] Vijai J (2013). Susceptibility loci associated with specific and shared subtypes of lymphoid malignancies. PLoS genetics.

[37] Wei S (2012). Genome-wide gene-environment interaction analysis for asbestos exposure in lung cancer susceptibility. Carcinogenesis.

[38] Jin F (2013). A high-resolution map of the three-dimensional chromatin interactome in human cells. Nature.

[39] Dong L, Pietsch S, Englert C (2015). Towards an understanding of kidney diseases associated with WT1 mutations. Kidney Int..

[40] Dong L (2015). Integration of Cistromic and Transcriptomic Analyses Identifies Nphs2, Mafb, and Magi2 as Wilms’ Tumor 1 Target Genes in Podocyte Differentiation and Maintenance. J Am Soc Nephrol.

[41] Kann M (2015). Genome-Wide Analysis of Wilms’ Tumor 1-Controlled Gene Expression in Podocytes Reveals Key Regulatory Mechanisms. J Am Soc Nephrol.

[42] Shibata S (2008). Modification of mineralocorticoid receptor function by Rac1 GTPase: implication in proteinuric kidney disease. Nat. Med..

[43] Ridley AJ, Paterson HF, Johnston CL, Diekmann D, Hall A (1992). The small GTP-binding protein rac regulates growth factor-induced membrane ruffling. Cell.

[44] Rottner K, Hall A, Small JV (1999). Interplay between Rac and Rho in the control of substrate contact dynamics. Curr. Biol..

[45] Weiger MC (2013). Real-time motion analysis reveals cell directionality as an indicator of breast cancer progression. PLoS One.

[46] Kaneto N (2014). RAC1 inhibition as a therapeutic target for gefitinib-resistant non-small-cell lung cancer. Cancer Sci..

[47] Li C, Wong WH (2001). Model-based analysis of oligonucleotide arrays: expression index computation and outlier detection. Proc. Natl. Acad. Sci. USA.

[48] Bolstad BM, Irizarry RA, Astrand M, Speed TP (2003). A comparison of normalization methods for high density oligonucleotide array data based on variance and bias. Bioinformatics.

[49] Quackenbush J (2001). Computational analysis of microarray data. Nature reviews. Genetics.

[50] Smyth GK (2004). Linear models and empirical bayes methods for assessing differential expression in microarray experiments. Stat. Appl. Genet. Mol. Biol..

[51] Storey JD, Tibshirani R (2003). Statistical significance for genomewide studies. Proc. Natl. Acad. Sci. USA.

[52] Saleem Ma (2002). A conditionally immortalized human podocyte cell line demonstrating nephrin and podocin expression. Journal of the American Society of Nephrology: JASN.

[53] Gee HY (2013). ARHGDIA mutations cause nephrotic syndrome via defective RHO GTPase signaling. The Journal of clinical investigation.

[54] Lennon R (2008). Hemopexin induces nephrin-dependent reorganization of the actin cytoskeleton in podocytes. Journal of the American Society of Nephrology: JASN.

[55] Guess A (2010). Dose- and time-dependent glucocorticoid receptor signaling in podocytes. Am. J. Physiol. Renal Physiol..

[56] Schiller BJ, Chodankar R, Watson LC, Stallcup MR, Yamamoto KR (2014). Glucocorticoid receptor binds half sites as a monomer and regulates specific target genes. Genome Biol..

[57] Reddy TE (2009). Genomic determination of the glucocorticoid response reveals unexpected mechanisms of gene regulation. Genome Res..

[58] Gibbs J (2014). An epithelial circadian clock controls pulmonary inflammation and glucocorticoid action. Nat. Med..

[59] Rostin M, Barthe P, Houin G, Alvinerie M, Bouissou F (1990). Pharmacokinetics of prednisolone in children with the nephrotic syndrome. Pediatr Nephrol.

[60] Miller PF, Bowmer CJ, Wheeldon J, Brocklebank JT (1990). Pharmacokinetics of prednisolone in children with nephrosis. Arch. Dis. Child..

[61] Grossmann C (2004). Transactivation via the human glucocorticoid and mineralocorticoid receptor by therapeutically used steroids in CV-1 cells: a comparison of their glucocorticoid and mineralocorticoid properties. European journal of endocrinology / European Federation of Endocrine Societies.

[62] Onesto C, Shutes A, Picard V, Schweighoffer F, Der CJ (2008). Characterization of EHT 1864, a novel small molecule inhibitor of Rac family small GTPases. Methods Enzymol..

