# Supplementary material for: Glucocorticoid therapy regulates podocyte motility by inhibition of Rac1
Source: Sci Rep. 2017 Jul 27;7:6725. doi: 10.1038/s41598-017-06810-y (PMC5532274; doi:10.1038/s41598-017-06810-y)
Supplement: Supplementary file 1 — Supplementary Information [file 41598_2017_6810_MOESM1_ESM.pdf]

Supplemental information

Glucocorticoid therapy alters podocyte motility by inhibition of Rac1

James C. McCaffrey (PhD)[1,2], Nicholas J Webb (MD),[2] Toryn Poolman(PhD),[3] Maryline Fresquet (MPhil),[1] Cressida Moxey,[1] Leo Zeef (PhD),[3] Ian J. Donaldson (PhD),[3] David W. Ray (PhD),[3] Rachel Lennon (PhD),[1,2]

1. Wellcome Trust Centre for Cell-Matrix Research, School of Biological Sciences, Faculty of Biology Medicine and Health, The University of Manchester 2. Department of Paediatric Nephrology, Royal Manchester Children's Hospital, Central Manchester University Hospitals NHS Foundation Trust, Manchester Academic Health Science Centre. 3. School of Medical Sciences, Faculty of Biology Medicine and Health, The University of Manchester.

Corresponding author:

Rachel Lennon

Wellcome Trust Centre for Cell-Matrix Research, Michael Smith Building, University of Manchester, Manchester, M13 9PT

Email: Rachel.Lennon@manchester.ac.uk

Table of Contents

1. Supplemental methods
2. Figure S1: The effect of puromycin aminonucleoside (PAN) treatment on podocytes
3. Figure S2: Effects of Gc and lipopolysaccharide (LPS) exposure on podocyte motility.
4. Figure S3: Effects of Gc on podocyte motility analysed using scratch wound assay
5. Figure S4: Rac1 activity following exposure to puromycin aminonucleoside (PAN).
6. Figure S5: Effect of Rac1 inhibition on podocyte motility.
7. Figure S6: Comparison of human podocyte ChIP-Seq dataset to other cell lines.
8. Figure S7: Full length western blots for Figure 1
9. Figure S8: Full length western blots for Figure 6
10. Table S1: Glucocorticoid-regulated genes involved with cellular motility

1. **Supplemental methods**

Immunofluorescence

Cells on coverslips were fixed in 4% (w/v) paraformaldehyde for 10 minutes at room temperature. Following two washed with PBS, cells were permeabilised with 0.5% (v/v) Triton X-100 in PBS for 10 minutes at room temperature, before blocking with 1% (w/v) bovine serum albumin (BSA) diluted in PBS for 10 minutes at room temperature. Coverslips were incubated with primary antibodies diluted in 1% BSA for 1 hour at room temperature. Coverslips were then washed three times with PBS before incubation with an appropriate secondary antibody diluted in 1% BSA for 1 hour at room temperature. After a further 3 washes with PBS, nuceli were stained with 4',6-diamidino-2-phenylindole (DAPI) before mounting onto glass slides using Polyvinyl alcohol mounting medium with DABCO® antifading reagent. Coverslips were imaged on a Delta Vision fluorescence microscope. Fluorescent images were acquired on a Delta Vision [RT] (Applied Precision) restoration microscope using a 40x/ numerical aperture 0.85 Uplan Apo objective and the Sedat filter set (86000v2). The images were collected using a Coolsnap HQ (Photometrics) camera with a Z optical spacing of 0.2μm. All images were taken with constant exposure time between all the conditions of the same staining. Images were processed using ImageJ 1.49c.

ChIP-Seq

Cell treatment and harvest

Twenty 15cm-diameter culture dishes of confluent wild-type human podocytes, approximating to $3x10^7$ cells, were used for each treatment condition. Twenty four hours prior to harvest, cell culture medium containing standard 10% fetal bovine serum (FBS) was exchanged with cell culture medium containing 10% charcoal-stripped FBS (Gibco™) to prevent endogenous Gc activating GR. One hour prior to harvesting, cells were treated with either 1μM prednisolone dissolved in methanol or an equal volume (0.001% v/v of media) of methanol alone as a vehicle control. Following treatment, cells were cross-linked in 0.1% (v/v) 11% formaldehyde solution for 10 minutes at room temperature, followed by quenching of the cross-linking reaction using 0.05% (v/v) 2.5 M glycine. Each plate was then washed twice with 1 x phosphate-buffered saline (PBS), before

cells were harvested using a cell scraper. Cells were pooled into a 50mL conical tube (separate tubes for Gc-treated and vehicle-treated samples) and centrifuged at 700x*g* for 5 minutes at 4°C. The supernatant was discarded and the cell pellet was resuspended in 10mL PBS. Tubes were centrifuged at 700x*g* for 5 minutes at 4°C and the supernatant was discarded. Samples were flash-frozen in liquid nitrogen and stored at -80°C.

Chromatin immunoprecipitation

Samples were processed using reagents from the ChIP-IT® High Sensitivity kit (Active Motif). Cell pellets from -80°C were resuspended in 5mL Chromatin Prep Buffer supplemented with 5µL of protease inhibitor cocktail (PIC) and 5µL 100nM phenylmethylsulfonyl fluoride (PMSF) at room temperature. Samples were homogenized using a Qiagen TissueRuptor® (power setting '4' for 45 seconds), before a 5 minute incubation on ice. Samples then underwent 90 stokes with a chilled Dounce homogeniser, before transfer to a 15 mL tube, which was centrifuged at 1250x*g* for 3 minutes at 4°C. The supernatant was discarded and the pellet was resuspended in 500µL ChIP Buffer supplemented with 5µL PIC and 5µL 100nM PMSF. Following a 10 minute incubation on ice, samples were sonicated using an Active Motif EpiShear® probe sonicator in combination with an EpiShear cooled sonication platform (35% power; 45 cycles- each cycle consisted of 30 seconds of sonication followed by 30 seconds of inactivity). Samples were then centrifuged at 4°C at maximum speed for 2 minutes to pellet the cellular debris. 100µL from the vehicle treated sample (for 'input' DNA) and 100µL from the +Gc sample were removed at this point to confirm that sonication produced appropriately sized DNA fragments. The remaining samples were stored in aliquots at -80°C. 100µL Tris-EDTA (TE) buffer, pH 8.0 and 2µL RNAase A (10ug/µL) were added to the 100µL chromatin samples before a 1 hour incubation at 37oC. 5µL proteinase K (10µg/µL) was then added before a 3 hour incubation at 37°C. The samples then underwent a 16 hour incubation at 65°C with 10µL 5M NaCl. DNA from the 100µL samples was purified using the Macherey-Nagel Nucleospin® kit. 1mL NTB was added to the sheared chromatin, and samples were spun through purification columns for 30 seconds at 11,000x*g*, and the flow-through was discarded. 700µL NT3 was then added to the columns before centrifugation for 30 seconds at 11,000x*g*, and the flow-through was

discarded. The step involving NT3 was performed twice, before the column was dried by 11,000x*g* spin for 1 minute. The column was then inserted into a new holding 1.5mL tube and 20µL NE buffer was added before a 1 minute incubation at room temperature. The column was centrifuged at 11,000x*g* for 1 minute. 18µL of the sample was placed into a PCR tube with 2µL 5M NaCl before a 20 minute incubation at 100°C, and the sonication efficiency was checked by running the sample on a 1.5% agarose gel (100 volts, 60 minutes). The chromatin aliquots stored at -80°C were thawed on ice, and spun at maximum speed at 4°C for 2 minutes. Overnight incubation at 4°C on an end-to-end rotator was performed with the following components: 205µL chromatin, 5µL blocker, 2µg Proteintech™ anti-human GR antibody (24050-1-AP) with 2µg of Sigma® anti- human GR antibody (HPA004248), and 5µL PIC (total volume 240µL). 30µL protein G agarose beads per reaction were washed twice in 30µL TE buffer. The chromatin/antibody mixture was centrifuged at 1250x*g* for 1 minute and 30µL protein G agarose beads were added for a 3 hour period at 4°C on an end to end rotator. ChIP reactions were centrifuged at 1250x*g* for 1 minute before 600µL ChIP buffer was added, and the mixture was transferred to a ChIP filtration column. The column was washed five times with 900µL AM1 wash buffer. The column was transferred to a 1.5mL tube before centrifugation for 3 minutes at 1250x*g*. Following transfer to a new 1.5mL tube, warm AM4 elution buffer was added to the column before centrifugation at 1250x*g* for 3 minutes. 2µL of proteinase K (10µg/µL) was added to the 100µL solution before a 30 minute incubation at 55°C. The cross-links were then reversed with a 2 hour incubation at 80oC. 500µL DNA purification binding buffer was added and the pH was adjusted to ensure pH<7.5 using 5µL aliquots of 3M sodium acetate. The sample was then added to the DNA purification column before centrifugation at 14,000 rpm for 1 minute, and the flow through was discarded. 750µL DNA purification wash buffer was added prior to another 14,000 rpm centrifugation for 1 minute. The column was dried with a 2 minute 14,000 centrifugation before transfer to a new 1.5mL tube. Warm purification elution buffer was added to the column before a 1 minute incubation at room temperature, followed by collection of purified ChIP DNA by centrifugation at 14,000rpm for 1 minute. Prior to sample sequencing, real-time polymerase chain reaction (qPCR) was performed to ensure enrichment of the +Gc samples over vehicle-treated samples for a known GR-binding site

(GBS) (*FKBP5+86*). Quantification of the concentration of DNA was performed using the Qubit™ fluorometer.

ChIP-Seq

Following Chromatin-immunoprecipitation (ChIP), DNA libraries were constructed according to the TruSeq® ChIP Sample Preparation Guide (Illumina). Briefly, sample DNA (5–10 ng) was blunt-ended and phosphorylated, and a single 'A' nucleotide added to the 3' ends of the fragments in preparation for ligation to an adapter with a single-base 'T' overhang. The ligation products were then purified and PCR-amplified to enrich for fragments with adapters on both ends. The final purified product was then quantitated prior to cluster generation on a cBot instrument and the loaded flow-cell then paired-end sequenced on an Illumina HiSeq2500 instrument. Data from the finished sequencing run was transferred to the Bioinformatics Core Facility (University of Manchester), on dedicated off-instrument storage space and converted to fastq formatted reads using the software CASAVA. Quality control was performed using FastQC v0.11.2. Read pairs (R1 and R2) were filtered using Trimmomatic v0.32 using paired-end mode, to remove adapters, and truncate reads (3') with a base sequence quality of <Q20, taken as an average of a 4bp moving window. Filtered reads <50b were removed. Default settings were applied. Filtered paired reads were mapped to human reference sequence (HG19/GRCh37; minus haplotypes) using Bowtie2 v2.2.3 with default parameters. Mapped paired-reads were filtered with 'samtools view' v0.1.19, to remove reads with mapping quality <Q30 and discordant pairs (i.e. incorrect orientation and/or >500bp apart). Only paired reads belonging to chromosomes 1-22, X and Y were used in downstream analyses (reads mapping to unassembled contigs and particularly the mitochondrial genome adversely affect the statistics generated by the peak caller). Regions of the genome significantly enriched with GR read-pairs, considered as fragments of DNA, compared to a background model using the input DNA fragments, were identified using model-based analysis for ChIP-Seq (MACS) 2 v2.1.0.20140616. Paired-end mapped reads enabled MACS2 to take the observed mean of DNA fragments, as opposed to approximating by cross-correlation when single-end reads are used. Binding regions were reported with a minimum q-value of 0.05, and fold enrichment was set at a cut-off of 5 over

background. The University of California, Santa Cruz (UCSC) human canonical genes for HG19 were associated with the summit regions (200bp centred on binding region summits) using RnaChipIntegrator (unpublished, by the BCF core facility). The two closest genes, by closest edges, up to 1 million bases away. Promoter region was designated as -2000b to 100b of transcriptional start site (TSS). Motif analysis was performed using HOMER.

Scratch wound assay

Fully differentiated wild-type podocytes were seeded on an Essen image lock 96 well plate, allowed to attach for 4 hours and then treated with 1µM prednisolone or vehicle. 18 hours following treatment, a manual scratch wound was made using a sterile pipette tip in each well. Media were replaced (to remove scratched/dead cells) with fresh media and treatments. Images were taken in each well to observe wound closure every 20 minutes for 12 hours. Two movies were filmed per well. Three wells were used per condition per treatment. The experiment was performed three times.

At time zero (T=0), 2 hours post-wounding (T=2), and 4 hours post-wounding (T=4), the area of the wound for each well was calculated using ImageJ software. The relative change in area from T=0 was determined using the equation: $\Delta A = (A_0 – A_n) / A_0$, where A_0 = area at T=0, A_n = area at time n, and ΔA is the relative change in area, as previously described.[12] Results were analysed using two-way ANOVA followed by Sidak’s multiple comparisons test.

Supplementary Figure S1

The effect of puromycin aminonucleoside (PAN) treatment on podocytes

Immortalised human podocytes were cultured in growth medium containing either vehicle or 5.0µg/mL of PAN. Immunofluorescence staining was performed using 4',6-diamidino-2-phenylindole (DAPI) for nuclear staining, and Texas Red-X phalloidin F-actin dye.

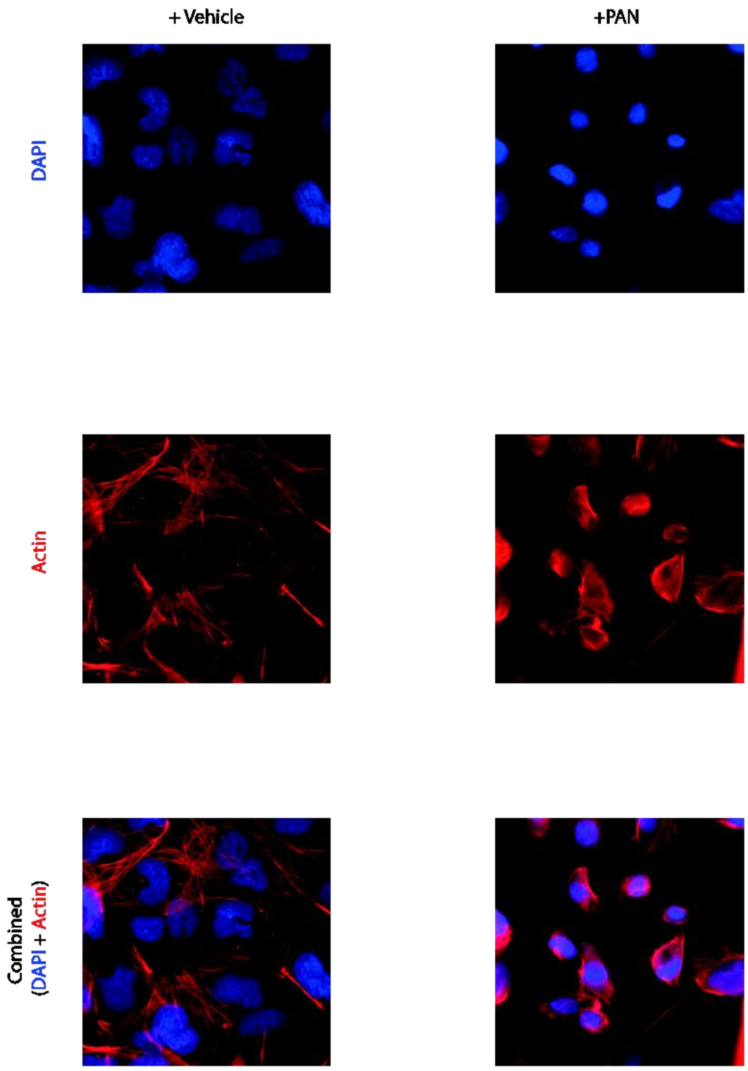

Supplementary Figure S2

<u>Effects of Gc and lipopolysaccharide (LPS) exposure on podocyte motility.</u>

Figure 4 showed analysis of effects of Gc and the podocyte-damaging agent PAN on podocyte motility. This figure shows analysis of the effects on podocyte motility of Gc and another podocyte-damaging agent, LPS. Again, live cell imaging was performed on WT podocytes for a 24 hour period, beginning 24 hours after treatment. Each experiment consisted of tracking 120 cells per condition. The experiment was performed three times. Manual tracking using ImageJ software was performed by a different individual for the PAN- and LPS-experiments to reduce the risk of observer bias. (A) quantifies mean podocyte speed over the 24 hour period. (B) quantifies podocyte directional persistence. Rose plots were created for each condition to visualize the path travelled for 20 cells (the path of each cell is marked with a different colour). The x- and y-axis refer to x,y coordinates of each cell over time generated by the cell tracking software: (C) vehicle-treated; (D) Gc-treated; (E) LPS-treated; (F) LPS+Gc-treated. (G) is a rose plot comparing the movement of 10 LPS-treated podocytes (red) compared with 10 podocytes treated with LPS+Gc (black) over the 24 hour period. Results were analysed by one-way ANOVA followed by Dunnett's multiple comparisons test. ****=adjusted p value <0.0001; ***= adjusted p value 0.0009; **=adjusted p value 0.0027. N.S.= not significant. Error bars represent standard error of the mean.

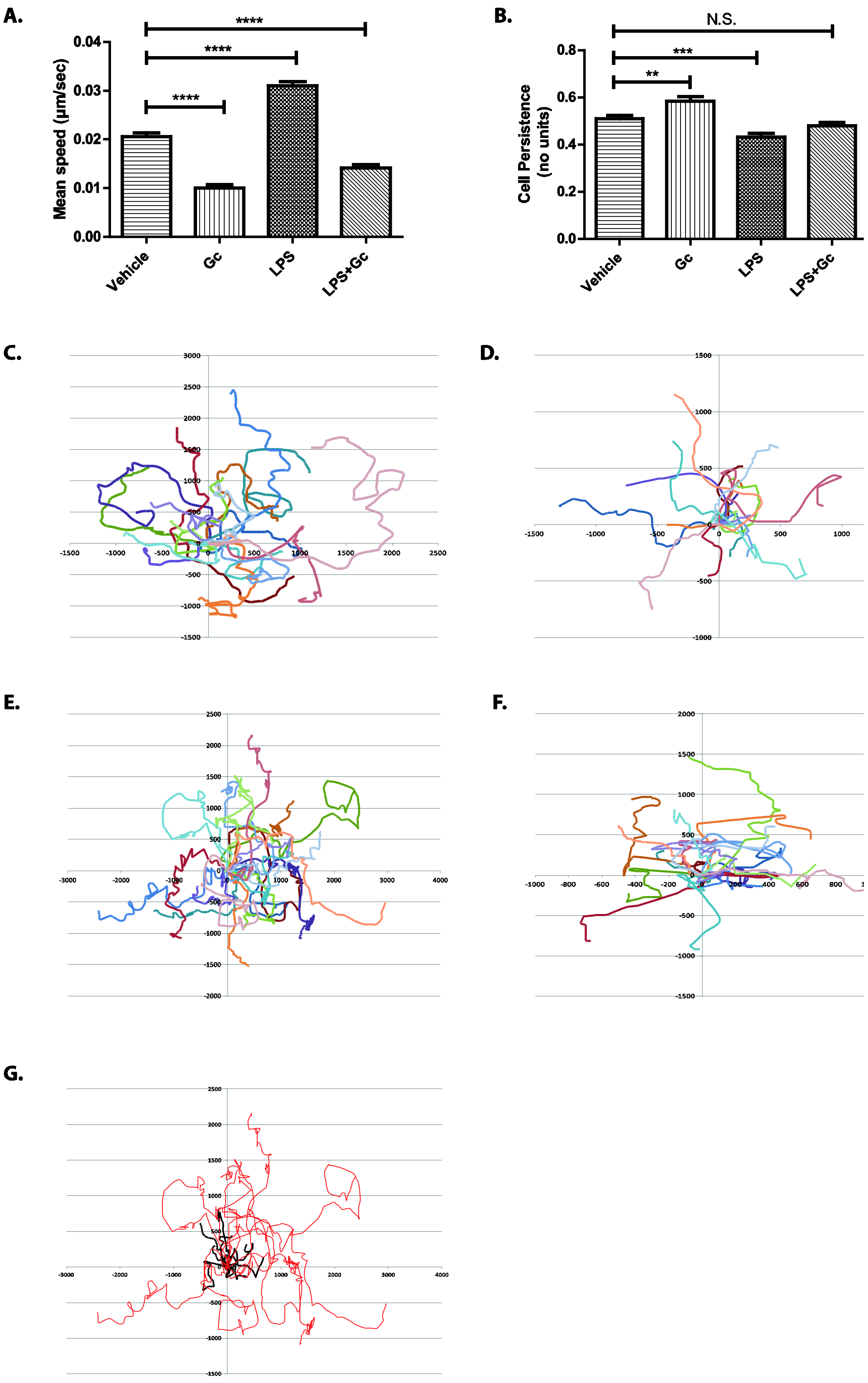
A.
Mean speed (µm/sec)
0.04
0.03
0.02
0.01
0.00
****
****
****
Vehicle
Gc
LPS
LPS+Gc
B.
Cell Persistence
(no units)
0.8
0.6
0.4
0.2
0.0
N.S.
***
**
Vehicle
Gc
LPS
LPS+Gc
C.
D.
E.
F.
G.

Supplementary Figure S3

Effects of Gc on podocyte motility analysed using scratch wound assay

A prominent reduction of podocyte motility following Gc exposure was observed using a cell-tracking technique. To validate this result, a scratch-wound assay was performed. A confluent monolayer of fully differentiated podocytes seeded into an Essen image lock 96 well plate were treated with either 1 µM Gc or vehicle. 18 hours after treatment, manual scratch wounds were made using sterile pipette tips. Media and treatments were replaced to remove scratched/dead cells and wound healing was filmed over a 12 hear period. Two movies were filmed per well. Three wells were used per condition per treatment. The experiment was performed three times. (A) shows representative images for vehicle- and Gc- treated wells at time zero (T=0), 2 hours post scratch wound and 4 hours post scratch wound. The area for each scratch wound was calculated using ImageJ software at time zero, 2 hours post scratch wound and 4 hours post scratch wound. The relative change in area from T=0 was determined using the equation: $\Delta A = (A_0 – A_n) / A_0$, where A_0 = area at T=0, A_n = area at time n, and ΔA is the relative change in area. Results were analysed using two-way ANOVA followed by Sidak's multiple comparisons test. (B) shows that at both time points (T = 4hrs and T = 6hrs), cells treated with vehicle showed a greater efficiency (0.34 for vehicle vs 0.16 for Gc-treated at T = 2hrs; 0.71 for vehicle vs 0.44 for Gc-treated at T = 4hrs). *** = p value <0.0001. Error bars represent standard error of the mean.

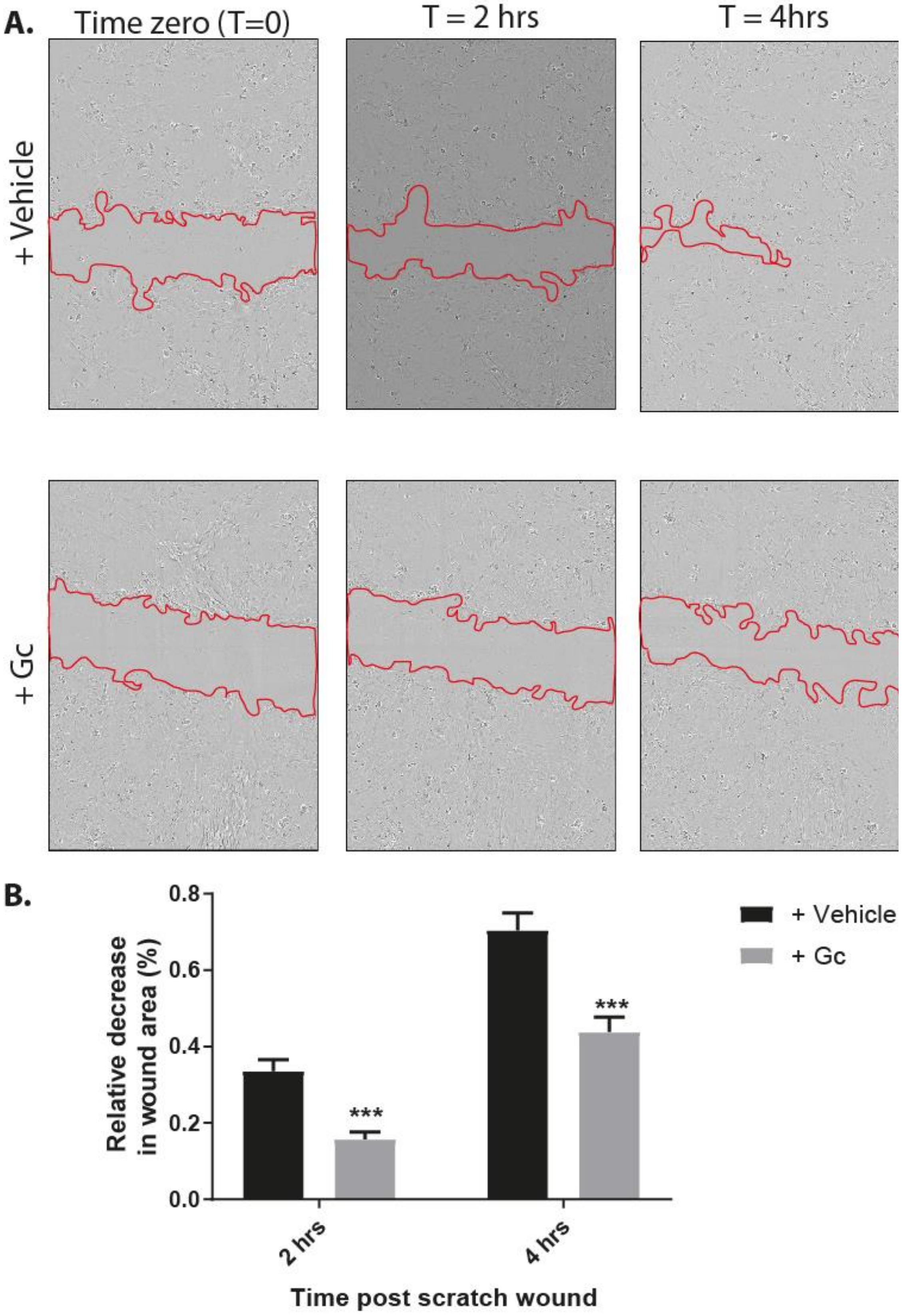
A.
Time zero (T=0)
T = 2 hrs
T = 4hrs
+ Vehicle
+ Gc
B.
Relative decrease in wound area (%)
0.0
0.2
0.4
0.6
0.8
***
***
2 hrs
4 hrs
Time post scratch wound
+ Vehicle
+ Gc

Supplementary Figure S4

Rac1 activity following exposure to puromycin aminonucleoside (PAN).

To understand if injuring podocytes with PAN affected Rac1-activity, pull-down assays using beads specific for active Rac1were performed following 24 hours of PAN exposure. This allowed quantification of the ratio of active Rac1 : total Rac1. (A) shows a representative western blot; (B) shows quantification. Results were analysed using the nonparametric Mann-Whitney U test. The experiment was performed four times. Error bars represent standard error of the mean.

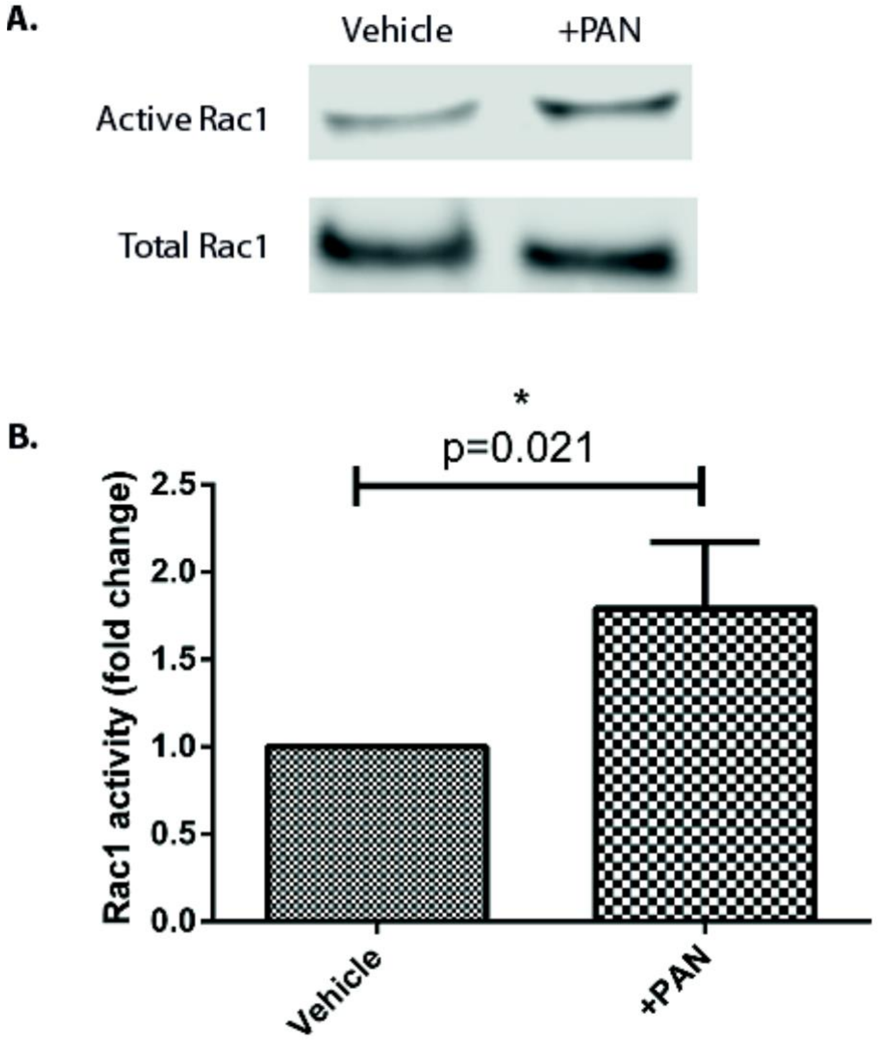

Supplementary Figure S5

Effect of Rac1 inhibition on podocyte motility

We had demonstrated that Gc exposure resulted in reduced podocyte speed and also reduced activity of the pro-migratory regulator Rac1. To investigate the effect of Rac1 inhibition on podocyte motility, we performed live cell imaging on wild type podocytes for a 36 hour period, beginning 2 hours after treatment with either vehicle or the specific Rac1 inhibitor EHT 1864. Each experiment consisted of tracking 80 cells per condition. The experiment was performed three times. Manual tracking was performed using ImageJ software and the mean speed of cells over the 36 hour period was quantified. Results were analysed using the nonparametric Mann-Whitney U test. **** = p value <0.0001.

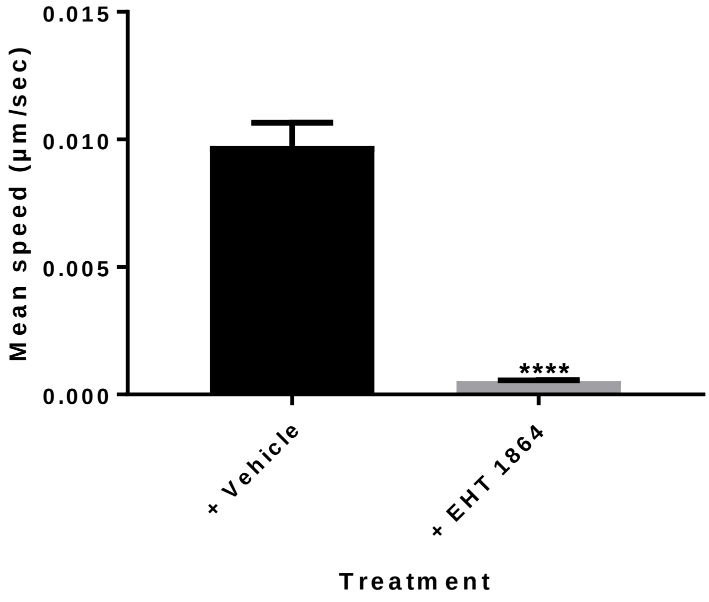

Supplementary Figure S6

Comparison of human podocyte ChIP-Seq dataset to other cell lines

In the current study, GR binding sites (GBS) were identified in human wild type podocytes using ChIP-Seq. The percentage of GBS found in particular genomic locations is shown in (A). For comparison, the location of GBS found in murine adipocytes (B),[24] rat pheochromocytoma (C),[25] and murine mammary adenocarcinoma cells (D),[26] from published studies are shown.

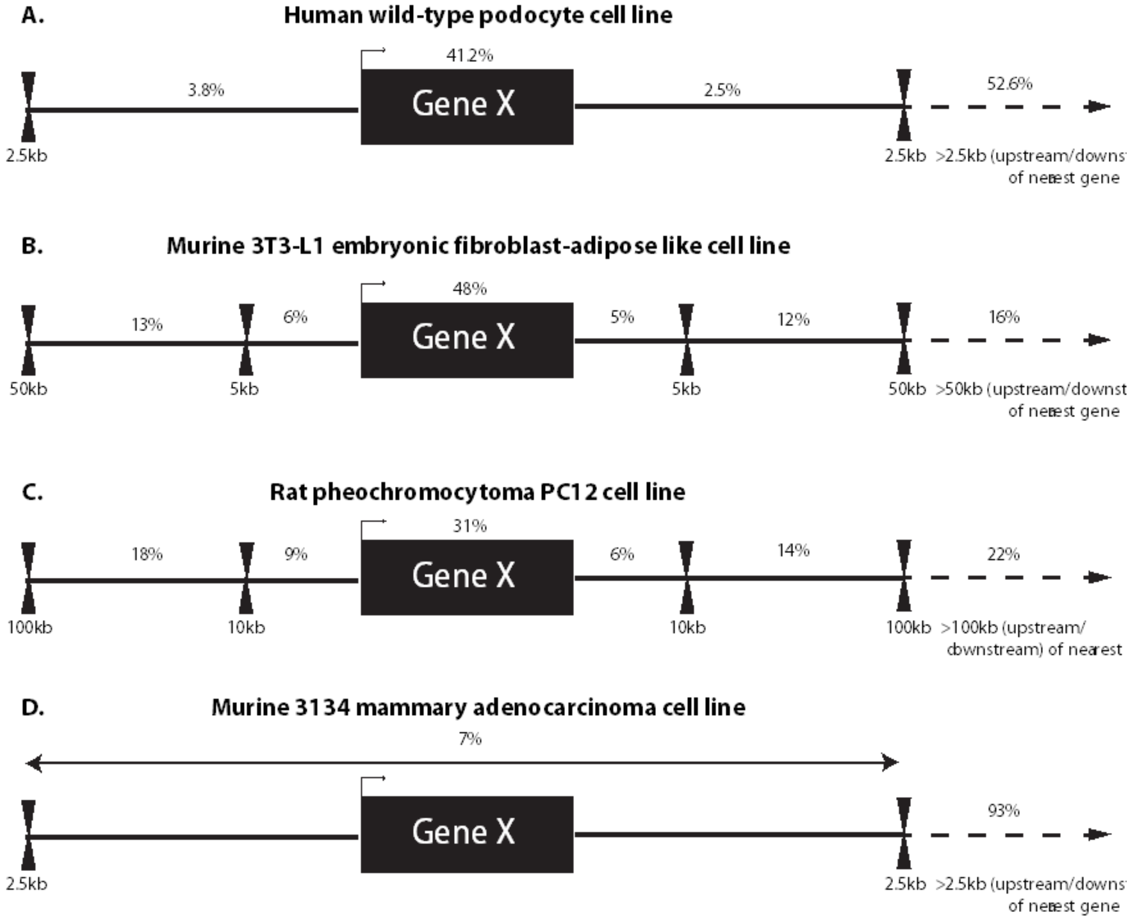

Supplementary Figure S7

Full length western blots

Full length blots corresponding to Figure 1. Indicated parts (red box) are shown in Figure 1 (d).

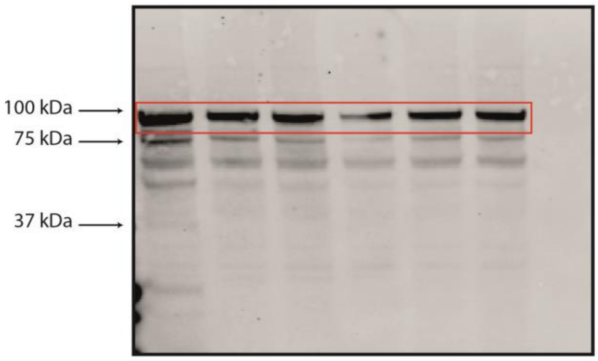

Glucocorticoid receptor (alpha isoform)

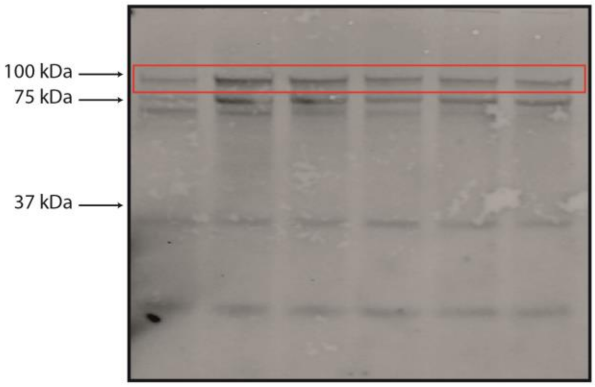

Phosphorylated glucocorticoid receptor α (Serine 211)

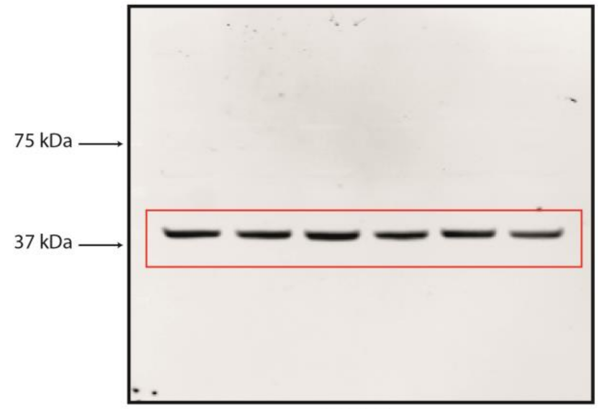

GAPDH

Supplementary Figure S8

Full length western blots

Full length blots corresponding to Figure 6. (a) Indicated parts (red box) are shown in Fig. 6a. (b) Indicated parts (red box) are shown in Fig. 6b. (c) Indicated parts (red box) are shown in Fig. 6c. (d) Indicated parts (red box) are shown in Fig. 6e.

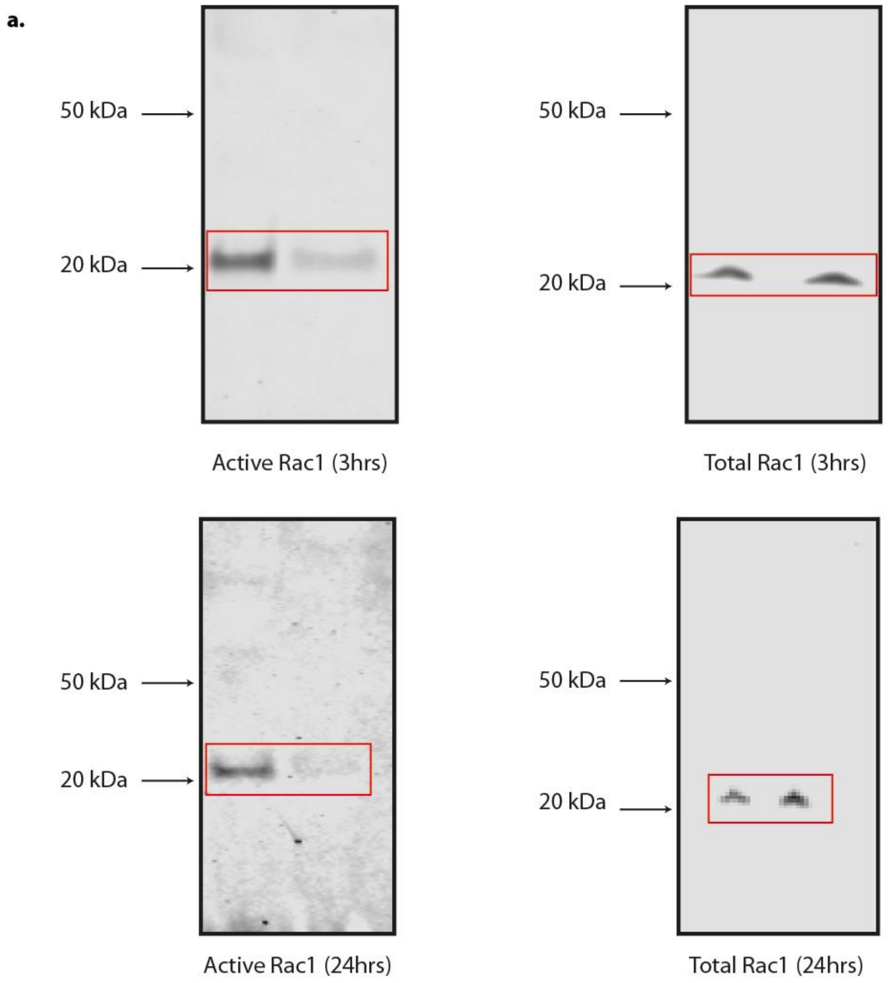

b.

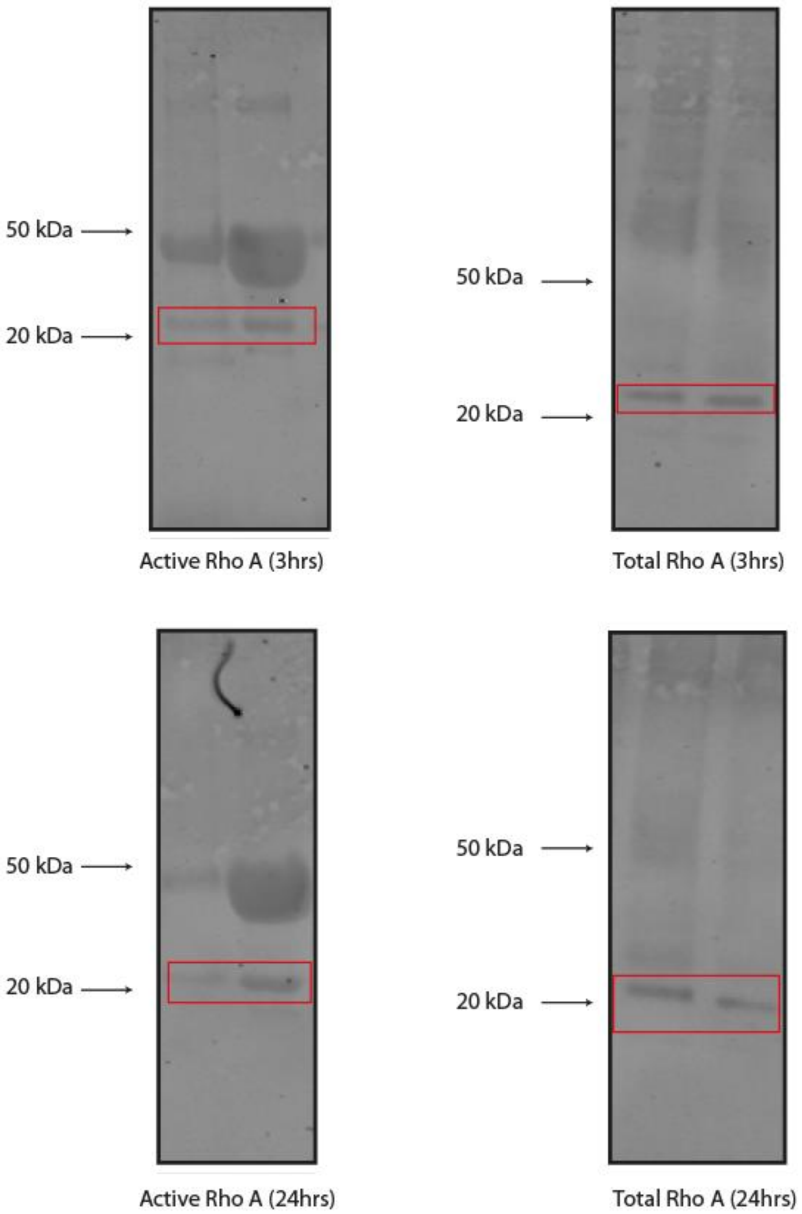
50 kDa
20 kDa
Active Rho A (3hrs)
50 kDa
20 kDa
Total Rho A (3hrs)
50 kDa
20 kDa
Active Rho A (24hrs)
50 kDa
20 kDa
Total Rho A (24hrs)

c.

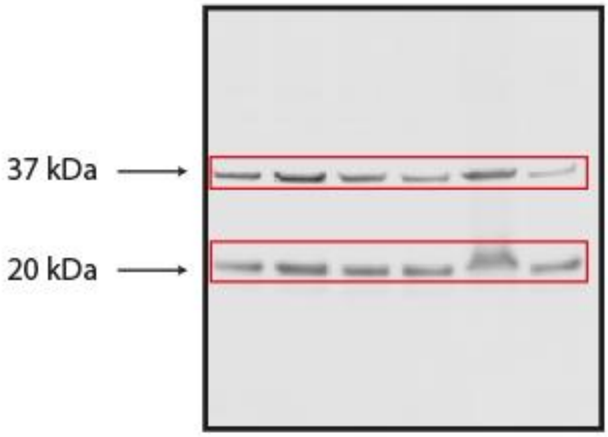

Rac1 and GAPDH

d.

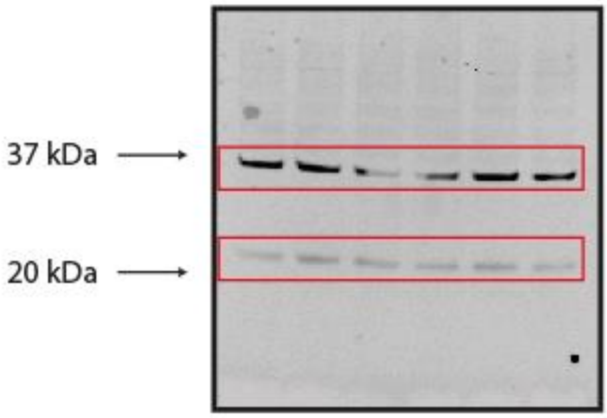

RhoA and GAPDH

Supplementary Table S1 Genes involved with cell movement

Genes from the microarray dataset identified using IPA software as having roles in cellular motility.

Genes in dataset	Predicted effect on cellular movement (based on expression direction)	Fold Change in gene expression following Gc-treatment
AREG	Increased	8.028
CCL20	Increased	4.375
TSC22D3	Decreased	4.339
IRS2	Increased	3.762
HPGD	Decreased	3.696
REPS2	Increased	3.498
CNR1	Increased	2.964
WNT5A	Increased	2.637
TGFBR3	Decreased	2.618
PER1	Affected	2.618
ANGPTL4	Increased	2.439
SCNN1A	Increased	2.37
LOX	Increased	2.363
TNFRSF21	Increased	2.363
FGD4	Affected	2.323
DCLK1	Increased	2.226
NRCAM	Affected	2.225
RGCC	Decreased	2.132

TGFBR1	Increased	2.069
COL4A3	Affected	2.063
ST3GAL6	Increased	2.036
SERPINE1	Increased	2.017
TNFSF4	Increased	1.975
DNAJB4	Decreased	1.967
PDE4DIP	Increased	1.95
EDN2	Increased	1.932
CCBE1	Increased	1.905
NDRG1	Decreased	1.891
NPR1	Increased	1.881
PRKX	Increased	1.869
IL1R1	Increased	1.85
HRH1	Increased	1.826
DUSP1	Decreased	1.794
NEDD9	Increased	1.789
MAP2	Increased	1.773
CEBPD	Increased	1.77
SLC1A3	Increased	1.751
TNFAIP3	Decreased	1.741
MYO9A	Decreased	1.73
DCN	Decreased	1.725
UNC5A	Increased	1.7

CD200	Decreased	1.688
GCNT1	Increased	1.684
DKK1	Decreased	1.621
EFNB2	Increased	1.616
ITGB6	Increased	1.608
CRYAB	Decreased	1.588
MALAT1	Increased	1.583
PTPN1	Decreased	1.581
APBB2	Affected	1.578
FOXC2	Increased	1.574
FPR1	Increased	1.57
PTGER4	Increased	1.568
KCNN4	Increased	1.559
LEF1	Increased	1.543
HIPK2	Increased	1.532
ST6GALNAC2	Increased	1.52
WWTR1	Increased	1.518
KLF4	Decreased	1.517
IL6ST	Increased	1.516
EPB41L5	Affected	1.503
GATA6	Decreased	-1.504
SHC4	Decreased	-1.523
EGR1	Decreased	-1.526

IL1B	Decreased	-1.535
SHC3	Affected	-1.544
GDF15	Increased	-1.546
KALRN	Decreased	-1.559
ACKR4	Decreased	-1.56
GDF5	Increased	-1.576
NR2F2	Affected	-1.579
ARID5B	Affected	-1.59
VEGFC	Decreased	-1.593
TNFRSF9	Decreased	-1.593
SPATA13	Affected	-1.595
TNFRSF19	Decreased	-1.596
FOXC1	Decreased	-1.601
BMP6	Decreased	-1.61
TFPI2	Increased	-1.629
CD274	Decreased	-1.67
FGF5	Decreased	-1.676
SEMA3A	Increased	-1.693
CSF2	Decreased	-1.702
CCL2	Decreased	-1.707
TM4SF1	Decreased	-1.711
GRIA3	Decreased	-1.712
PLAU	Decreased	-1.749

SERPINB2	Decreased	-1.774
SPRY2	Increased	-1.8
VCAM1	Decreased	-1.827
GDNF	Decreased	-1.859
JAG1	Increased	-1.866
TBX3	Decreased	-1.902
ADAMTS1	Decreased	-1.905
TNFSF10	Affected	-1.945
HBEGF	Decreased	-1.949
NOG	Decreased	-1.958
PTHLH	Decreased	-2.015
ASB2	Decreased	-2.076
NPPB	Increased	-2.104
TRIB1	Affected	-2.176
CYP1B1	Affected	-2.28
TNFRSF11B	Decreased	-2.361
IL1A	Decreased	-2.57
IL11	Decreased	-3.365
